# Nrf2/PHB2 alleviates mitochondrial damage and protects against *Staphylococcus aureus*‐induced acute lung injury

**DOI:** 10.1002/mco2.448

**Published:** 2023-12-07

**Authors:** Si‐Hao Jin, Jiao‐Jiao Sun, Gang Liu, Li‐Juan Shen, Yuan Weng, Jin‐You Li, Min Chen, Ying‐Ying Wang, Zhi‐Qi Gao, Feng‐Juan Jiang, Sheng‐Peng Li, Dan Chen, Qing‐Feng Pang, Ya‐Xian Wu, Zhi‐Qiang Wang

**Affiliations:** ^1^ Department of Cardiothoracic Surgery Affiliated Hospital of Jiangnan University Wuxi China; ^2^ Department of Basic Medicine, Wuxi School of Medicine Jiangnan University Wuxi China; ^3^ Department of Nursing, School of Medicine Shaoxing Vocational & Technical College Shaoxing China; ^4^ Department of Nosocomial Infection The Forth Affiliated Hospital of Zhejiang University Jinhua China; ^5^ Department of Critical Care Medicine Wuxi Hospital of Traditional Chinese Medicine Wuxi China; ^6^ Department of Laboratory Affiliated Hospital of Jiangnan University Wuxi China

**Keywords:** acute lung injury, mitochondria, nuclear factor E2‐related factor 2, prohibitin 2, sepsis, *Staphylococcus aureus*

## Abstract

*Staphylococcus aureus* (SA) is a major cause of sepsis, leading to acute lung injury (ALI) characterized by inflammation and oxidative stress. However, the role of the Nrf2/PHB2 pathway in SA‐induced ALI (SA‐ALI) remains unclear. In this study, serum samples were collected from SA‐sepsis patients, and a SA‐ALI mouse model was established by grouping WT and Nrf2^−/−^ mice after 6 h of intraperitoneal injection. A cell model simulating SA‐ALI was developed using lipoteichoic acid (LTA) treatment. The results showed reduced serum Nrf2 levels in SA‐sepsis patients, negatively correlated with the severity of ALI. In SA‐ALI mice, downregulation of Nrf2 impaired mitochondrial function and exacerbated inflammation‐induced ALI. Moreover, PHB2 translocation from mitochondria to the cytoplasm was observed in SA‐ALI. The p‐Nrf2/total‐Nrf2 ratio increased in A549 cells with LTA concentration and treatment duration. Nrf2 overexpression in LTA‐treated A549 cells elevated PHB2 content on the inner mitochondrial membrane, preserving genomic integrity, reducing oxidative stress, and inhibiting excessive mitochondrial division. Bioinformatic analysis and dual‐luciferase reporter assay confirmed direct binding of Nrf2 to the PHB2 promoter, resulting in increased PHB2 expression. In conclusion, Nrf2 plays a role in alleviating SA‐ALI by directly regulating PHB2 transcription and maintaining mitochondrial function in lung cells.

## Graphical abstract

In this study, the authors investigated that activation of the Nrf2/PHB2 pathway in alveolar epithelial type II cells (AT II) can alleviate mitochondrial damage caused by *Staphylococcus aureus*‐induced sepsis in mice, thereby promoting the survival of AT II and preventing progression to acute lung injury. Nrf2 plays a key role in the mitochondrial retention of PHB2, and its nuclear translocation can increase the expression of PHB2 to prevents mitochondrial dysfunction

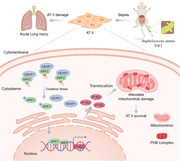

## 1 INTRODUCTION

*Staphylococcus* aureus (*S. aureus*, SA) is an opportunistic and dangerous Gram‐positive pathogen.^1^ In case where *S. aureus* is not effectively eliminated from the host and persists in bloodstream colonization, it culminates in the development of severe sepsis. Furthermore, the persistent presence of inflammation triggered by *S. aureus* can result in a cascade of immune responses, ultimately leading to a cytokine storm and contributing to the development of acute lung injury (ALI).^2^ Antibiotic therapy is the only option to treat *S. aureus* infectious diseases, but the widespread emergence of drug‐resistant species such as methicillin‐resistant *S. aureus* comes with a huge set of challenges.^3^, ^4^ The development of alternative strategies to combat *S. aureus* infections has become imperative. Increasing recognition of the role played by oxidative damage and inflammation as key factors in the pathogenesis of SA‐induced ALI (SA‐ALI).^5^ However, the molecular mechanisms by which SA‐induces oxidative damage in lung tissue are still unknown.

Mitochondria serve as a crucial link between various modes of cell death, including autophagy, apoptosis, and ferroptosis.^6^, ^7^ The role of mitochondria and their associated cellular pathways is known to undergo alterations in the context of sepsis. During the progression of sepsis, the mitochondrial electron transport chain is impaired in multiple organs, resulting in increased free radical production, oxidative phosphorylation (OXPHOS) defects, and reduced adenosine triphosphate (ATP) generation.^8^ These dysfunctions contribute to the disruption of mitochondrial homeostasis. Furthermore, mitochondrial dysfunction leads to apoptosis of alveolar type II epithelial (AT‐II) cells and disruption of the epithelial barrier, which is a physical barrier that protects the alveoli from environmental damage by isolating inhaled foreign substances and regulating the transport of water and ions.^9^, ^10^, ^11^

Nuclear factor E2‐related factor 2 (Nrf2) has been demonstrated to mitigate mitochondrial dysfunction and sepsis‐induced ALI through elucidated mechanisms. Nrf2 activates antioxidant response elements (ARE), well established as significant regulators of redox homeostasis and activators of cytoprotection during oxidative stress, that regulate the expression of more than 200 protective genes such as heme oxygenase 1 and NAD(P)H quinone dehydrogenase 1 following its dissociation from Kelch‐like ECH‐associated protein 1 (Keap1) and nuclear translocation.^12^, ^13^, ^14^ In addition to regulating cellular redox homeostasis, Nrf2 has been also reported to be involved in mitochondrial biogenesis, the process of producing new functional mitochondria, and which could restore mitochondrial function after various stimuli or injuries, through activation of ARE/eroxisome proliferator‐activated receptor‐gamma coactivator 1α (PGC‐1α) signaling pathway.^15^ Activation of Nrf2 can maintain mitochondrial integrity by promoting mitophagy and conferring resistance to oxidative stress‐mediated permeability transition, to reduce cell death.^16^ The mitochondrial membrane potential (MMP) is a universal indicator of mitochondrial health and the metabolic state of the cell. Nrf2 alters basal MMP and substrate consumption rate and oxygen consumption. Compared with wild type (WT), basal state oxygen consumption was 50 and 35% lower in Nrf2 or Keap1 deletion due to impaired mitochondrial complex I activity.^17^ The use of malate/pyruvate, which in turn increase the production of the complex I substrate NADH, or methyl succinate, a substrate for complex II, resulted in a higher rate of MMP rise in cells with Nrf2 upregulation than in WT.^18^

Prohibitin 2 (PHB2) is a highly conserved protein that is expressed in the mitochondria, nucleus, and plasma membrane of many cell types.^19^ Most of the cellular effects exerted by PHB2 are related to its regulatory role in mitochondria.^20^ PHB2 regulates a variety of mitochondrial functions^21^ including mitophagy activation,^22^ mitochondrial biogenesis,^23^ and crista morphogenesis.^24^ Nrf2 expression was shown to be positively correlated with that of PHB2 in a rat model of subarachnoid hemorrhage.^25^ This implies a potential synergistic interplay between Nrf2 and PHB2 in regulating cellular responses to oxidative stress and mitochondrial dysfunction, thereby providing a basis to speculate that Nrf2 might augment mitochondrial function, mitigate oxidative stress, and ameliorate sepsis‐induced ALI through the modulation of PHB2. However, further investigations are warranted to elucidate the specific mechanisms and implications of the interplay between Nrf2 and PHB2 in SA‐ALI. Continued exploration in this regard will contribute to unraveling the precise roles of Nrf2 and PHB2 in disease progression and provide novel insights for the development of targeted therapeutic strategies against SA‐ALI.

In this study, we tested the hypothesis that Nrf2 activation promotes PHB2 transcription to maintain mitochondrial function and alleviate septic ALI caused by *S. aureus*, along with the underlying mechanisms using in vitro and in vivo models.

## 2 RESULTS

### 2.1 Decreased Nrf2 expression in *S. aureus*‐induced sepsis patients

**FIGURE 1:**
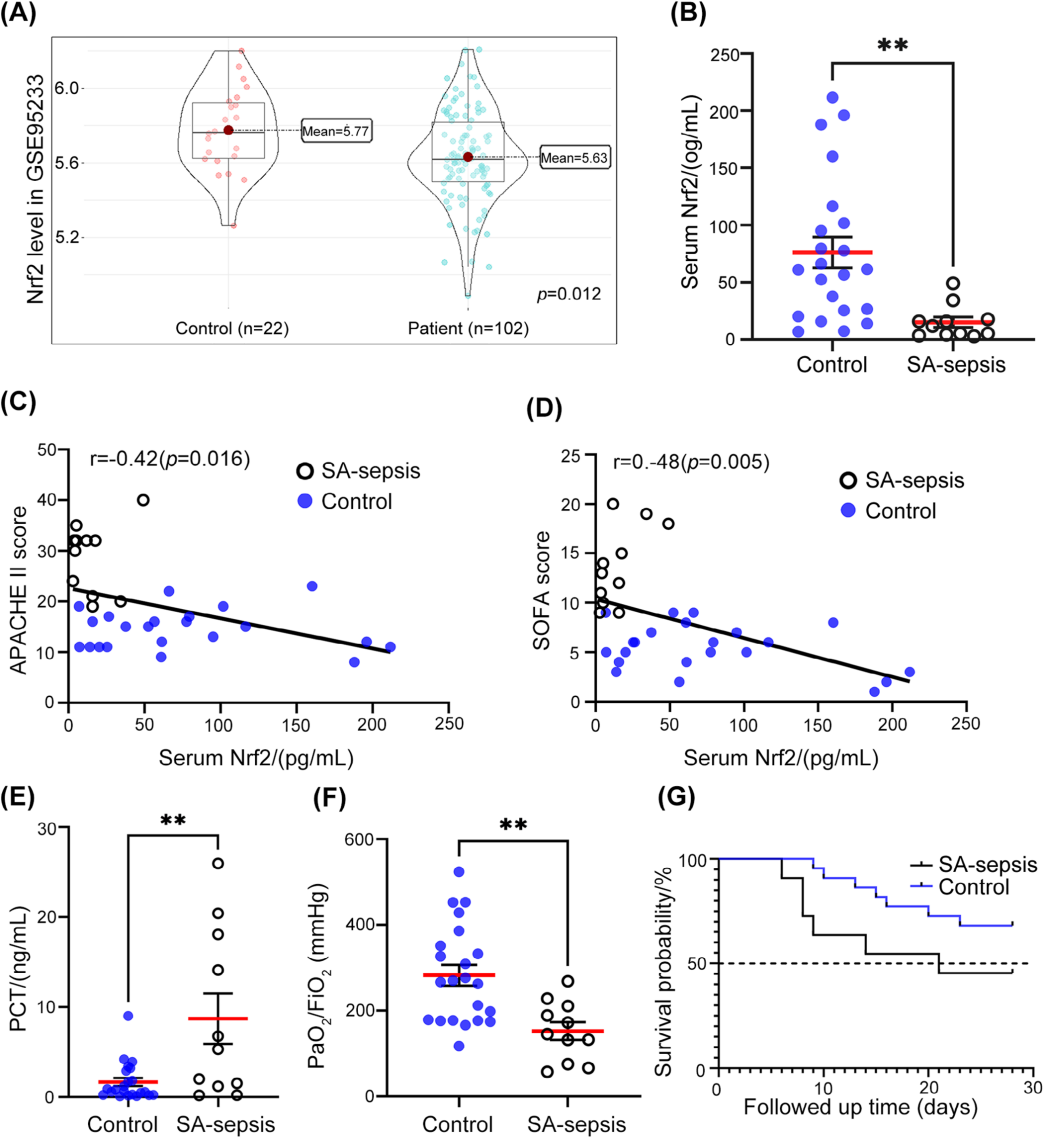
Decreased Nrf2 expression in *S. aureus*‐induced sepsis (SA‐sepsis) patients. (A) Gene expression of Nrf2 in 22 healthy samples and 102 sepsis samples were obtained from GSE95233. (B) Nrf2 protein levels in serum from control (*n* = 22) and SA‐sepsis (*n* = 11) patients. (C and D) Correlation between serum Nrf2 content and APACHE II/SOFA score in SA‐sepsis. (E and F) Levels of PCT and PaO_2_/FiO_2_ from the clinical data of patients. (G) The survival probability of patients within 28 days after admission to the ICU was analyzed. **p < 0.01.

To investigate the role of Nrf2 in *S. aureus*‐induced sepsis (SA‐sepsis), we first analyzed gene expression data from patients with septic shock caused by *S. aureus* infection.^26^ The transcriptomes of peripheral whole blood from healthy volunteers (control, *n* = 22) and septic shock patients (patient, *n* = 102) were analyzed, and they showed separate clustering patterns (Figure S1A). Additionally, gene expression levels were found to be similar within each subject (Figure S1B). Nrf2 mRNA level was lower in septic shock patients than in controls (*p =* 0.012; Figure 1A).

To evaluate the potential clinical relevance of Nrf2 in SA‐sepsis, we compared the levels of Nrf2 in serum samples from patients with sepsis and intensive care unit (ICU) patients without sepsis. We observed significantly lower Nrf2 levels in the SA‐sepsis group (Figure 1B). The results revealed a negative correlation between serum Nrf2 levels and the Acute Physiology and Chronic Health Evaluation II (APACHE II) score (*r* = −0.42, *p =* 0.016; Figure 1C) as well as the Sequential Organ Failure Assessment (SOFA) score (*r* = −0.48, *p =* 0.005; Figure 1D). Additionally, the SA‐sepsis group exhibited more severe organ damage compared with the control group. Meanwhile, serum level of procalcitonin (PCT)—a marker of bacterial infection^27^—was much higher in the SA‐sepsis group than in the control group (*p =* 0.002; Figure 1E and Table 1). The lower PaO_2_/FiO_2_ ratio in the SA‐sepsis group (*p =* 0.002; Figure 1F) indicated that lung function was impaired in sepsis patients. Approximately 81.8% of patients in the SA‐sepsis group had ALI, which was significantly higher than the proportion of control patients (*p =* 0.026; Table 1). Although there was no difference in 28‐day survival between the 2 groups (*p =* 0.121; Figure 1G), the mortality rate of the SA‐sepsis was higher than that of the control (58.3 vs. 31.8%; Table 1). Thus, the downregulation of Nrf2 in SA‐sepsis leads to multiple organ dysfunction, with the expression level of Nrf2 being negatively correlated with sepsis severity.

**TABLE 1:** Patient demographics for *S. aureus*‐induced sepsis and control patients.

	*S. aureus*‐induced sepsis patients (*n* = 11)	Control patients (*n* = 22)	Inspection value (*t*, *χ* ^2^,or *z*)	*p* Value
Age (range)	79.4 ± 6.5 (65–92)	75.1 ± 9.9 (50–91)	1.294	0.205
Sex (%)	Male = 7 (63.6%)	Male = 16 (72.7%)	0.287	0.696
Chronic comorbidities				
Diabetes	7	9	1.517	0.282
Coronary atherosclerosis	3	6	0.000	>0.999
Hepatic insufficiency	2	1	1.650	0.252
Heart failure	1	2	0.000	>0.999
Hypertension	9	11	3.110	0.132
Renal insufficiency	2	1	1.650	0.252
COPD	2	1	1.650	0.252
Clinical data				
Shock	4	3	1.843	0.211
Acute lung injury (%)	9 (81.8%)	8 (36.4%)	6.066	0.026
Acute kidney injury (%)	7 (63.6%)	6 (27.3%)	4.062	0.065
Blood/sputum culture results				
*S. aureus* isolates only	9	0		
Others	0	11		
Mixed bacterial isolates	2	1		
Bacterial growth status in sputum culture				
(+)	2			
(++)	5			
(+++)	4			
Bacterial growth status in blood culture				
Negative	7			
Positive	4			
Site of infection				
Pneumonia	11	17		
Abdominal infection	0	1		
Skin and soft tissue infection	0	5		
Catheter‐related infection	0	2		
Physiological parameters during exposure window				
PCT [ng/mL; IQR]	5.3 [1.19, 18.08]	0.72 [0.24, 2.98]	−1.988	0.047
CRP (mg/L; range)	105.9 ± 97.9 (8.7–335.9)	58.5 ± 60.8 (3.1–271.0)	1.718	0.096
Serum lactate concentration [mg/dL; IQR]	1.6 [1.00, 2.20]	1.4 [0.98, 2.60]	‐0.623	0.533
MAP [mmHg; IQR]	83.67 [77.67, 92.33]	80.00 [75.58, 94.75]	‐0.255	0.799
PaO_2_/FiO_2_ (mmHg)	152.39 ± 69.09 (57.0–268.9)	282.60 ± 113.06 (117.1–524.2)	3.492	0.002
Creatinine [μmol/L; IQR]	129.00 [87.40, 246.00]	117.70 [62.48, 194.63]	−1.172	0.241
APACHE II score (range)	28.8 ± 6.8 (19–40)	14.5 ± 4.0 (8–23)	7.627	<0.001
SOFA score (range)	13.6 ± 4.0 (9–20)	5.5 ± 2.4 (1–9)	7.453	<0.001
Mechanical ventilation (%)	10 (90.91%)	18 (81.8%)	0.471	0.643
ICU length of stay (d; IQR)	14 [8, 21]	20.5 [14.5, 34.75]	−1.123	0.261
Death within 28 days (%)	7 (58.3%)	7 (31.8%)	3.039	0.136

Variables were presented as mean ± SD, number of patients (*n*) or median [IQR]. (+), (++), and (+++) represent the *S. aureus* concentration ranging from 10^5^ to 10^6^ CFU/mL, 10^6^ to 10^7^ CFU/mL, and 10^7^ to 10^8^ CFU/mL, respectively. COPD, chronic obstructive pulmonary disease; PCT, procalcitonin; CRP, C‐reactive protein; MAP, mean arterial pressure; APACHE II, acute physiology and chronic health evaluation II; SOFA, sequential organ failure assessment; IQR, interquartile range.

The results revealed a downregulation of Nrf2 expression in patients with septic shock caused by *S. aureus* infection. Lower levels of Nrf2 were associated with more severe organ damage and showed a negative correlation with the severity scores of sepsis.

### 2.2 Differentially expressed genes and enrichment analysis

Whole‐genome transcriptome analysis of blood samples from septic shock and control patients revealed 1023 differentially expressed genes (DEGs), including 607 upregulated and 416 downregulated genes (Figure S1C; *p* < 0.05). The top 50 DEGs are presented in a heatmap (Figure S2). Many of the top DEGs (Figure S2) are known to be involved in redox reactions (*TXN* and *ATP5F1E*), regulation of mitochondrial function (*NDUFA1*, *ATP5F1E*, *ATP5MG*, and *ATP5MJ*), and immune regulation (*MCEMP1*, *IRAK3*, *FCER1G*, *LILRA5*, *IL1R2*, and *IL18RAP*).

**FIGURE 2:**
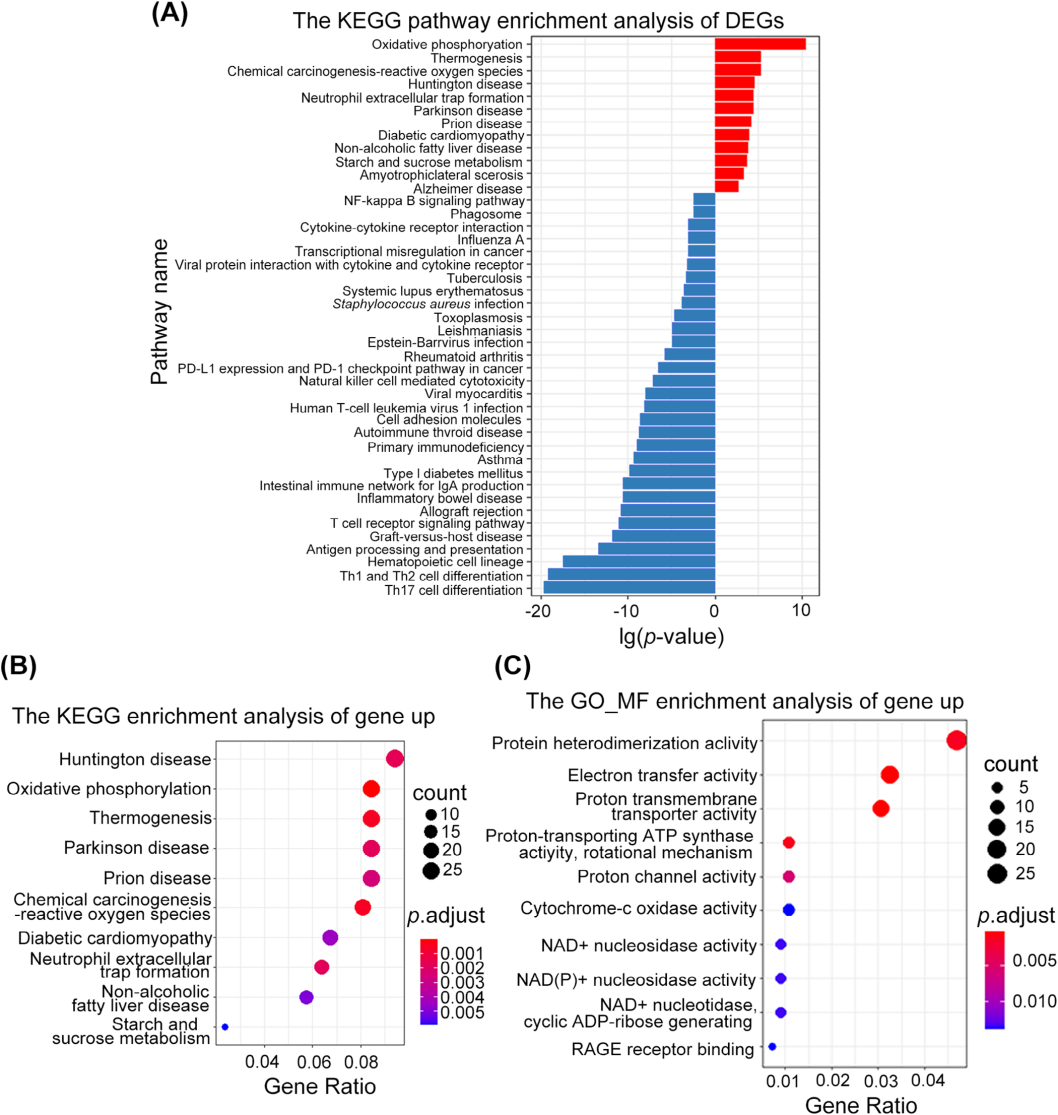
Differentially expressed genes and enrichment analysis. (A) The KEGG (https://www.kegg.jp/kegg) pathway enrichment analysis of DEGs in GSE95233. (B) The top 10 KEGG pathways enrichment analysis of up regulate genes in GSE95233. (C) Top 10 enrichment terms in the molecular function (MF) in control and sepsis patients.

To identify molecular functions and signaling pathways that are significantly altered in sepsis, we performed gene ontology (GO) and Kyoto encyclopedia of genes and genomes (KEGG) pathway enrichment analyses. KEGG pathways associated with all DEGs (Figure 2A) and upregulated genes (Figure 2B) suggested that OXPHOS (KO00190) plays an important role in sepsis. Consistent with these results, GO molecular function terms for genes upregulated in sepsis were enriched in OXPHOS‐related functions such as electron transfer activity, cytochrome‐c oxidase activity, and NAD^+^ nucleosidase activity (Figure 2C). These results indicate that altered OXPHOS and mitochondrial function play an important role in the development of sepsis.

Transcriptomic analysis revealed 1023 DEGs in patients with septic shock, which are potentially associated with oxidative‐reduction reactions, mitochondrial function, and immune regulation (Figure S2). Enrichment analysis highlighted significant alterations in the OXPHOS pathway (Figures 2A and B), may suggesting the importance of disrupted mitochondrial function in the development of sepsis.

### 2.3 Occurrence of oxidative stress injury and mitochondrial dysfunction during SA‐ALI in mice

**FIGURE 3:**
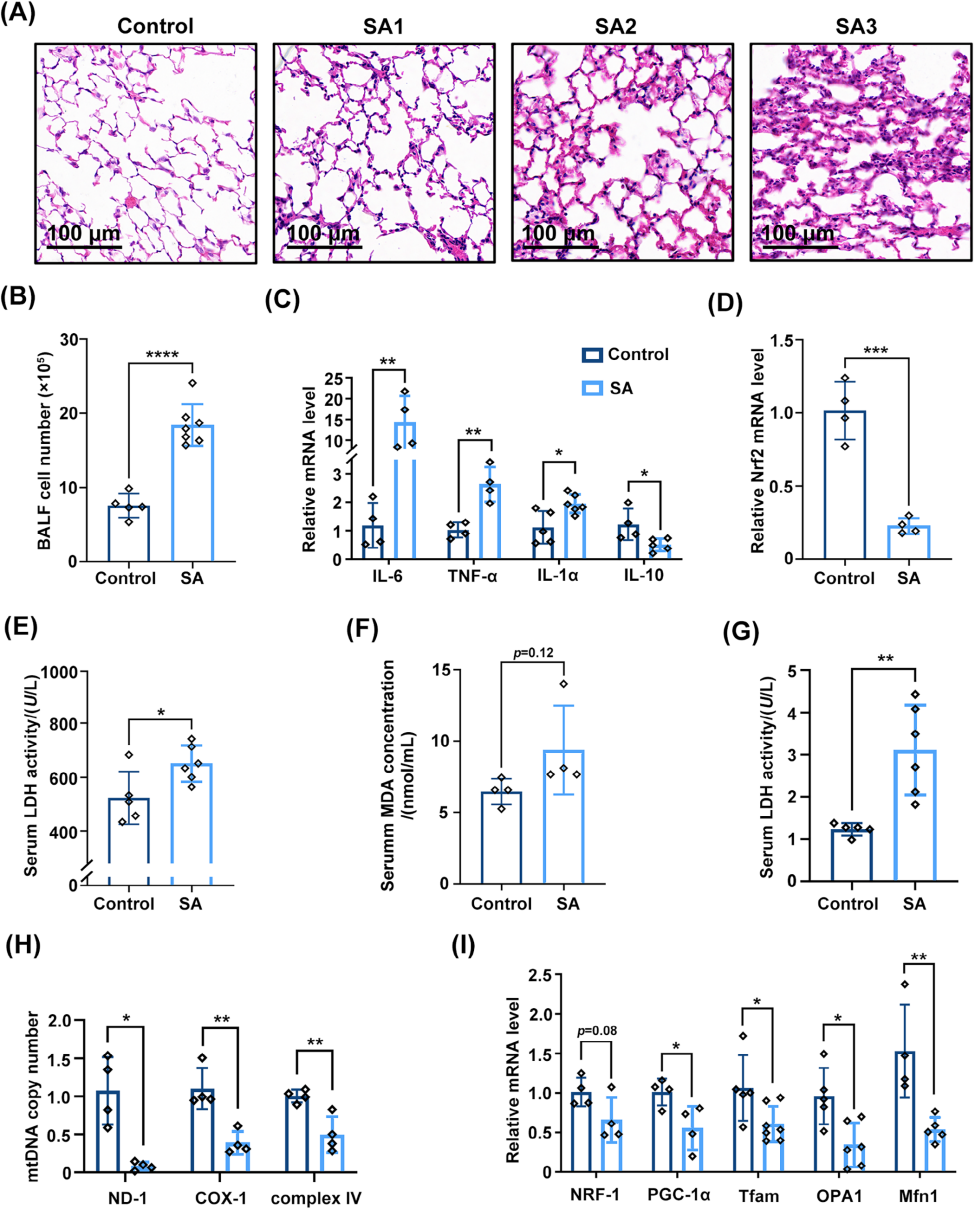
Occurrence of oxidative stress injury and mitochondrial dysfunction during SA‐ALI in mice. (A) Structural alteration and inflammatory infiltration of lung tissues after sepsis using H&E staining (magnification, ×100, bar = 100 μm; *n* = 5). SA1, 2 × 10^7^ CFUs *S. aureus*; SA2, 1 × 10^8^ CFUs *S. aureus*; SA3, 3 × 10^8^ CFUs *S. aureus*. (B) Cell counting of BALF from mice was performed by flow cytometry. (C, D, and I) RNA was isolated from the lung tissue of mice with sepsis caused by *S. aureus*. RT‐qPCR was performed to determine the mRNA level. (E–G) LDH, MDA, and MPO activities were measured in mice serum. (H) The relative levels of ND‐1 and COX‐1 were used to reflect the mtDNA copy number. Experiments were repeated at least three times. *p < 0.05, **p < 0.01, ***p < 0.001.

To further examine the role of Nrf2 in SA‐ALI, a SA‐ALI model was established using WT C57BL/6 mice (Figure S3A). Hematoxylin and eosin (H&E) staining of lung tissue revealed that with increasing *S. aureus* concentration, edema, necrosis, and alveolar and interstitial inflammation were aggravated compared with the control group (Figure 3A). The alveolar structure was largely destroyed after being injected with the highest concentration of bacteria. Bronchoalveolar lavage fluid (BALF) cell counts revealed that inflammatory cells had infiltrated the lung tissue of SA‐ALI mice (*p* < 0.05; Figure 3B); and real‐time quantitative PCR (RT‐qPCR) analysis showed that interleukin (IL)−6, IL‐1α, and TNF‐α were upregulated while IL‐10 was downregulated in this group (Figure 3C). In line with the findings from clinical samples, Nrf2 mRNA expression was much lower in the SA‐ALI group (*p* < 0.001; Figure 3D). Serum lactate dehydrogenase (LDH), malondialdehyde (MDA), and myeloperoxidase (MPO) activities were also significantly increased in the sepsis model (Figures 3E–G).

Mitochondrial function is thought to play a critical role in the development of ALI in sepsis.^28^ *PGC‐1α*, mitochondrial transcription factor A (*Tfam*) and nuclear respiratory factor 1 (*NRF1*) are implicated in mitochondrial biogenesis triggered by environmental stressors,^29^ while mitofusin 1 (*MFN1*) and optic atrophy 1 (*OPA1*) are involved in mitochondrial fusion.^30^ We found that mitochondrial DNA (mtDNA) copy number was decreased in the lung tissue of SA‐ALI mice (Figure 3H). Additionally, it was accompanied by downregulation of *PGC‐1α*, *Tfam*, *NRF1*, *MFN1*, and *OPA1* (Figure 3I) mRNA, indicating that mitochondrial biogenesis and fusion were inhibited at the transcriptional level during *S. aureus* infection. Thus, sepsis caused by *S. aureus* leads to ALI and is accompanied by oxidative stress injury and mitochondrial dysfunction.

Using a SA‐ALI mouse model, we observed increased lung damage, inflammation, and altered cytokine expression. Nrf2 mRNA expression was significantly lower in the SA‐ALI group, and markers of oxidative stress and inflammation were elevated. Additionally, mitochondrial dysfunction markers and reduced mitochondrial biogenesis and fusion‐related gene expression were detected, indicating the occurrence of oxidative stress injury and mitochondrial dysfunction during SA‐ALI in mice.

### 2.4 Nrf2 deficiency aggravates SA‐ALI with mitochondrial dysfunction

**FIGURE 4:**
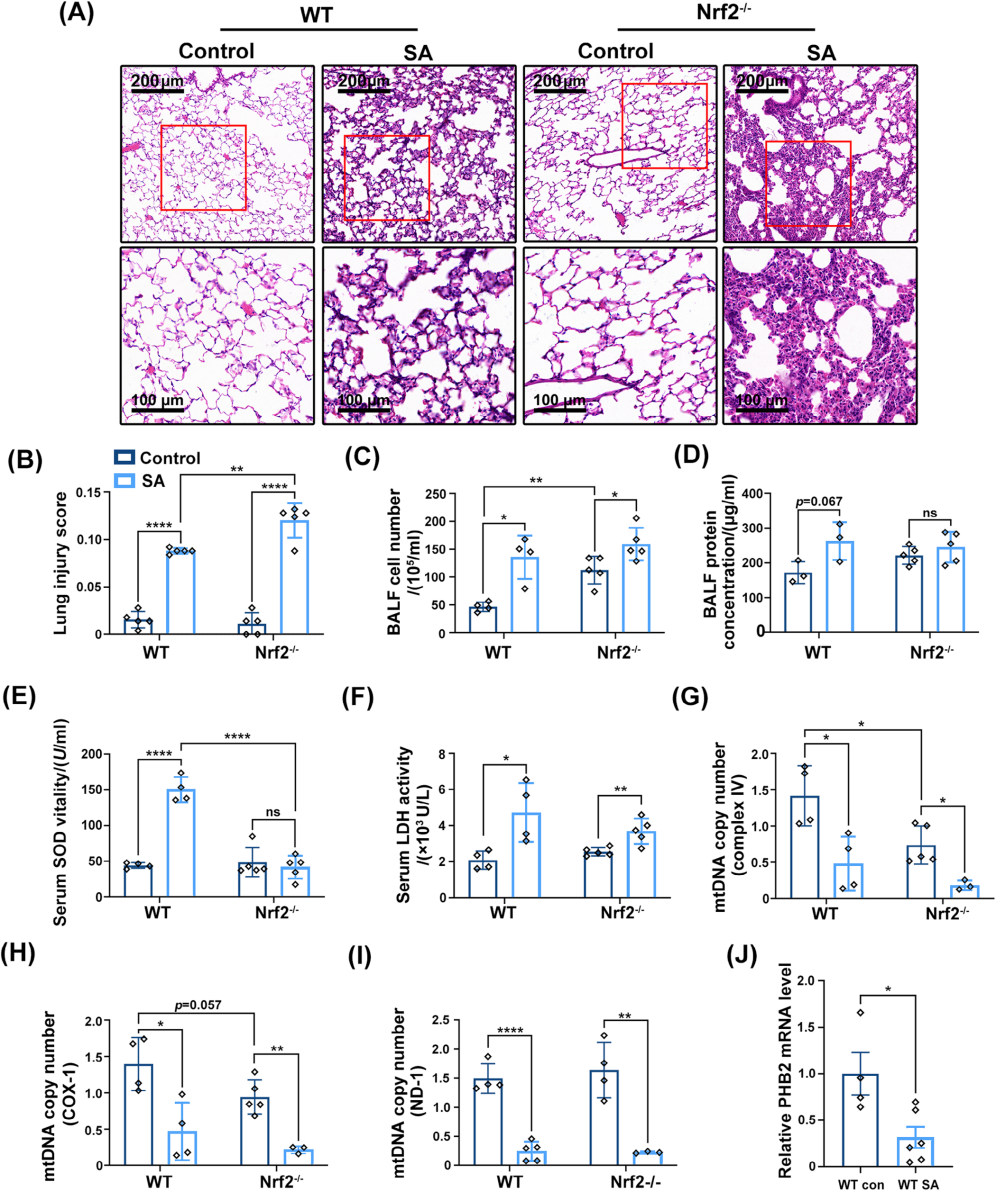
Nrf2 deficiency aggravates SA‐ALI with mitochondrial dysfunction. (A) Pathological changes in lung tissue due to sepsis and Nrf2 deficiency. (magnification, ×100 and ×400, bar = 200 and 100 μm; *n* = 5). (B) Lung injury scores based on the pathological results. (C and D) Cell counts and protein concentration in BALF from mice. (E and F) Levels of serum SOD and LDH from Nrf2^−/−^ mice. (G–I) The relative mRNA levels of complex IV, COX‐1, and ND‐1 were used to reflect the mtDNA copy number. (J) PHB2 mRNA level in lung tissue. Experiments were repeated at least three times. *p < 0.05, **p < 0.01, ****p < 0.0001, ns denotes not significant.

We examined whether loss of Nrf2 aggravates SA‐ALI using Nrf2^−/−^ mice (Figures S3B and C). H&E staining of lung tissue indicated that inflammatory cell infiltration was more severe in Nrf2^−/−^ mice than in the WT (Figure 4A). In Nrf2^−/−^ mice with *S. aureus* infection, alveolar walls were thickened and contained numerous neutrophils; alveoli showed structural damage; and many inflammatory cells had infiltrated around the trachea (Figure 4A). The lung injury score showed that Nrf2^−/−^ mice had severe lung tissue damage (*p =* 0.0045; Figure 4B). The number of cells in BALF was increased in sepsis and Nrf2 deficiency increased inflammatory cell infiltration (Figure 4C). However, there was no significant difference in BALF protein concentration between Nrf2^−/−^ and WT mice (Figure 4D). SA‐ALI mice also showed a marked increase in serum superoxide dismutase (SOD), which can convert superoxide anion radicals into two new form—oxygen and H_2_O_2_,^31^ but the antioxidant enzyme defense system was not activated in the absence of Nrf2 (Figure 4E). The change in serum LDH level was consistent with our previous results (Figure 3E), but Nrf2 knockout did not alter the level of LDH compared with the WT (Figure 4F). An examination of mtDNA copy number showed that loss of Nrf2 further inhibited mitochondrial function in sepsis (Figures 4G and H) as well as in healthy mice (Figure 4I).

In addition to Nrf2, another regulator of mitochondrial functions is PHB2. We evaluated PHB2 mRNA level and found that it was decreased when under the attack of *S. aureus* in vivo (Figure 4J). Consistent with our experimental results, PHB2 also showed a decreasing trend in septic shock patients (Figure S3D).

Collectively, the results showed that Nrf2 deficiency exacerbated lung tissue damage, inflammatory cell infiltration, and mitochondrial dysfunction in SA‐ALI. Additionally, the mRNA level of PHB2, another regulator of mitochondrial functions, was decreased in both mice and septic shock patients.

### 2.5 Nrf2 inhibition exacerbates mitochondrial dysfunction in vitro

**FIGURE 5:**
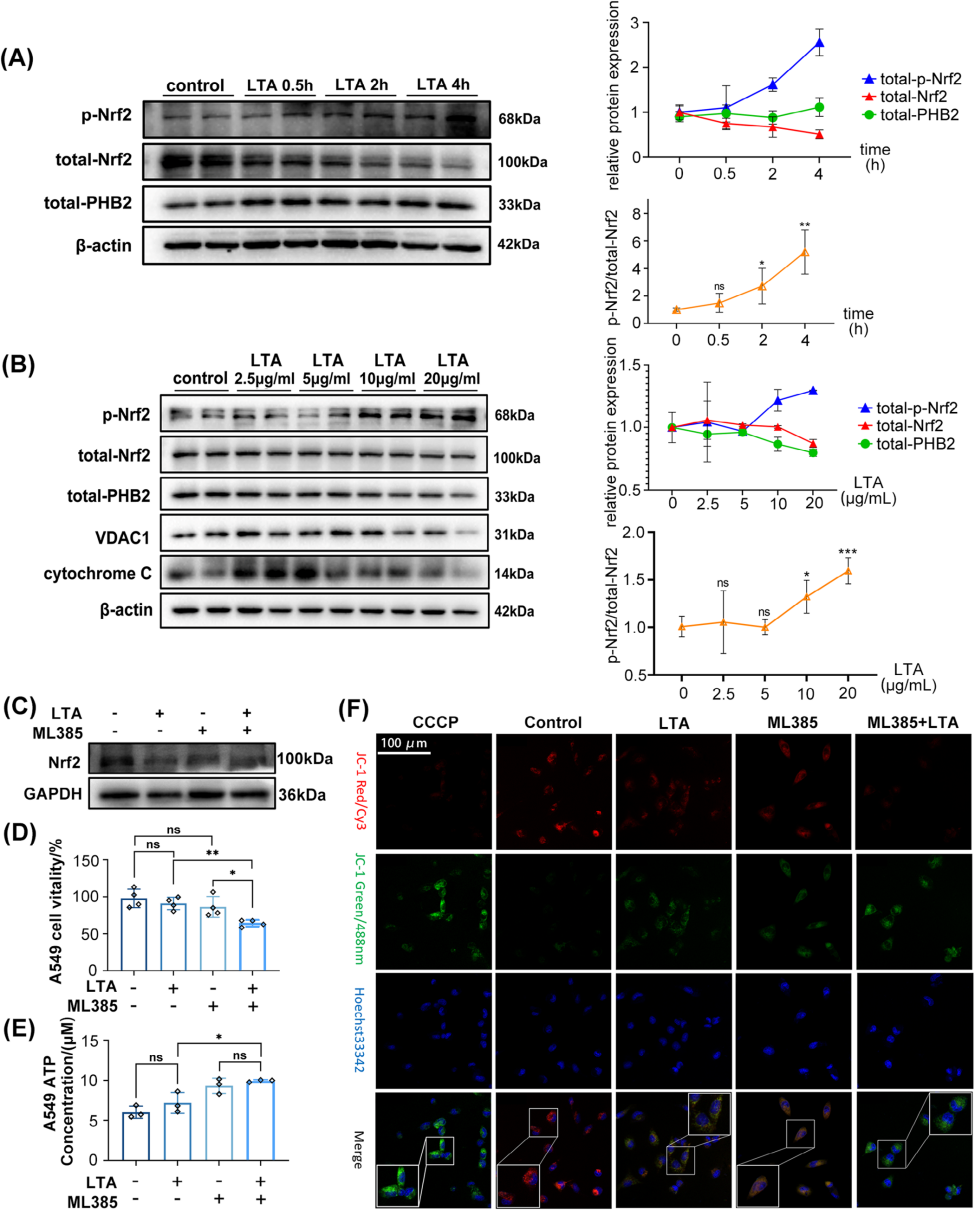
Nrf2 inhibition exacerbates mitochondrial dysfunction in vitro. (A) In vitro, total proteins were collected from LTA‐treated A549 cells at concentrations of 10 μg/mL for 30 min, 2 h, and 4 h, respectively. (B) A549 cells were treated separately by LTA at different concentration gradients for 4 h. β‐Actin were used as internal reference proteins. (C) Nrf2 level in cells after ML385 and LTA treatment was detected using western blot. (D and E) Cell vitality and ATP concentration was measured in A549 cells. (F) MMP was recorded using JC‐1 staining (magnification, ×400, bar = 100 μm; *n* = 3). Experiments were repeated at least three times. *p < 0.05, **p < 0.01, ns denotes not significant.

To explore the mechanism of SA‐ALI in greater detail, we used A549 cells treated with lipoteichoic acid (LTA), a major component of the *S. aureus* cell wall. Western blot analysis showed that Nrf2 expression decreased with prolonged LTA (10 μg/mL) treatment, especially after 4 h, whereas PHB2 level was unchanged (Figure 5A). We also examined phospho‐Nrf2 (p‐Nrf2) expression with LTA time gradients and concentrations, and found that to be expressed more with prolonged LTA treatment and increased concentration (Figures 5A and B). However, the expression of total‐Nrf2 (t‐Nrf2) is opposite to that of p‐Nrf2. The ratio of p‐Nrf2/t‐Nrf2 showed an increasing trend as the concentration of LTA and treatment time increased. When cells were treated with higher concentrations of LTA (20 μg/mL) for 4 h, Nrf2 and PHB2 levels were decreased, which also reduced voltage‐dependent anion channel 1 (VDAC1) expression (Figure 5B). Cytochrome *c* has an antioxidant function in cells and is a marker of mitochondrial function.^32^ Treatment with a low concentration of LTA (2.5 μg/mL) resulted in the release of a large amount of cytochrome *c*—a reactive oxygen species scavenger and initiator of the apoptosis cascade—from mitochondria. However, intracellular cytochrome *c* was depleted at a high concentration (20 μg/mL) of LTA (Figure 5B).

To clarify the role of mitochondria in the effects exerted by Nrf2, the Nrf2 inhibitor ML385 was used in this study (Figure 5C). Neither ML385 (10 μM) nor LTA (20 μg/mL) affected cell vitality but cotreatment with both agents had an inhibitory effect, as determined with the cell vitality assay (Figure 5D). There was no significant difference in ATP production following treatment with ML385 and LTA, but there was an increasing trend overall (Figure 5E). MMP—which drives ATP synthesis and is a key parameter for evaluating mitochondrial function^33^—was reduced by LTA and ML385 separately, as determined using the MMP probe JC‐1 (Figure 5F). Moreover, the coapplication of LTA and ML385 synergistically enhanced this effect (Figure 5F). These results indicate that Nrf2 inhibition aggravates mitochondrial impairment in lung cells.

Our experiments demonstrated that as the treatment duration and concentration of LTA increased, the expression level of Nrf2 decreased in vitro. Additionally, the expression level of PHB2 decreased with an increase in the concentration of LTA treatment. Nrf2 inhibition, along with LTA treatment, led to reduced cell viability and MMP, indicating that Nrf2 inhibition exacerbates mitochondrial dysfunction in lung cells.

### 2.6 Nrf2 modulates PHB2 expression and intracellular distribution during SA‐ALI

**FIGURE 6:**
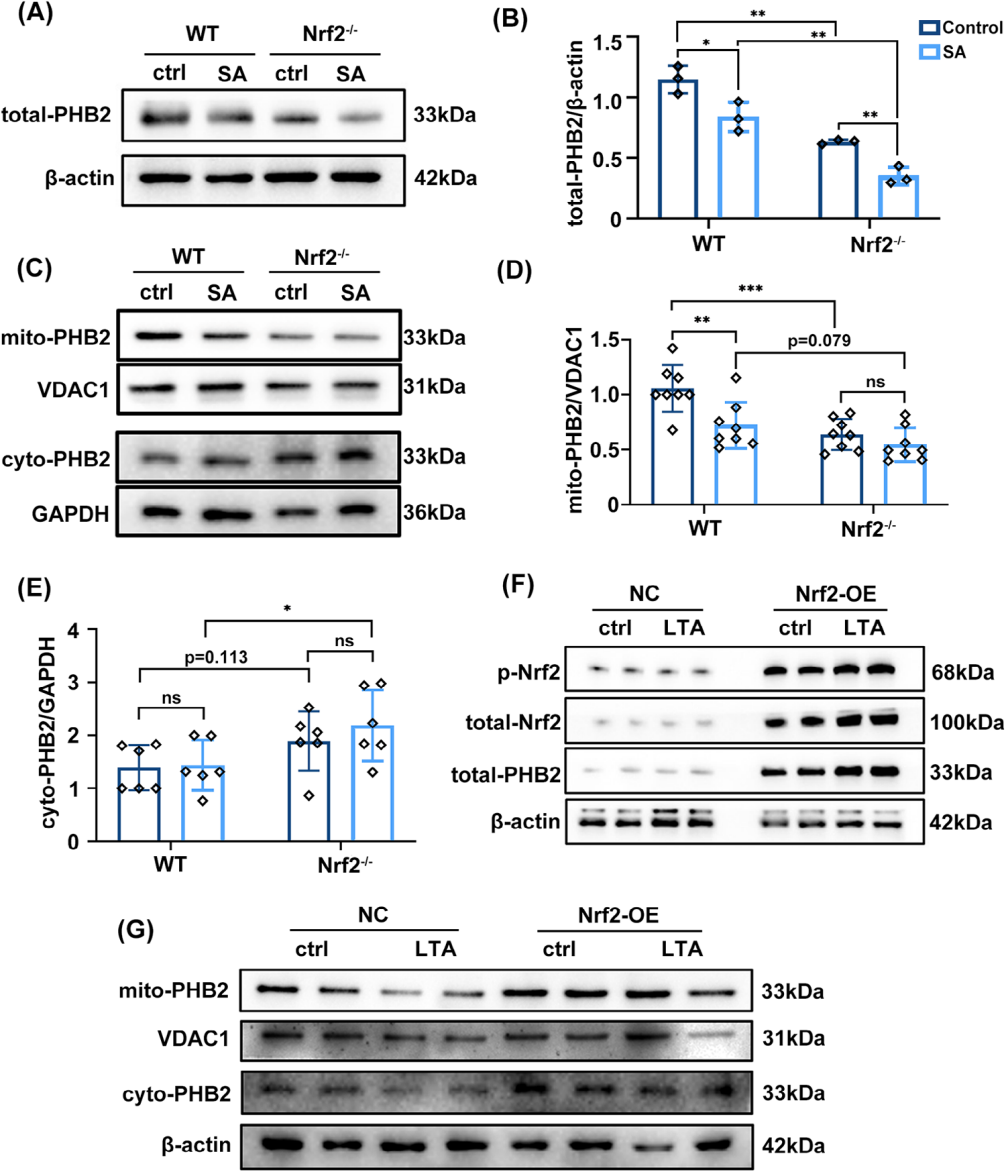
Nrf2 modulates PHB2 expression and intracellular distribution during SA‐ALI. (A and C) In vivo, mitochondrial and cytosolic fractions were collected from lung tissues. Western blots were used to determine the expression of PHB2. VDAC1 and GAPDH was utilized as the loading control for mitochondrial and cytosolic fraction, respectively. (B, D, and E) Quantitative analysis of bands for total‐, mito‐ and cyto‐PHB2. (F) A549 cells were transfected with Nrf2‐OE or empty (NC) plasmid for 48 h. Total Nrf2 and PHB2 protein expression were determined by western blot. (G) Cyto‐ and mito‐PHB2 were measured in A549 cells. Experiments were repeated at least three times. *p < 0.05, **p < 0.01, ***p < 0.001, ns denotes not significant.

To examine the intracellular distribution of PHB2 in SA‐ALI, we evaluated PHB2 protein levels in different cellular compartments. Our results show that the expression of total‐PHB2 in lung tissues of WT mice decreased under *S. aureus* infection, and the absence of Nrf2 further reduced the expression of total‐PHB2 (Figures 6A and B). Subsequently, we performed mitochondrial and cytoplasmic fractionation in lung tissue. Western blot analysis showed that mitochondrial PHB2 (mito‐PHB2) level was decreased in Nrf2^−/−^ mice and was further reduced by SA‐ALI (Figures 6C and D). In contrast, Nrf2 deficiency combined with SA‐ALI led to an increase in cytoplasmic PHB2 (cyto‐PHB2) level (Figures 6C and E). Immunofluorescence analysis revealed a decrease in PHB2 expression following LTA and ML385 treatment (Figure S4). Thus, during SA‐ALI, both intracellular and mitochondrial levels of PHB2 were decreased, while the cytoplasmic level of PHB2 was significantly increased, and these effects was enhanced in the absence of Nrf2. Subsequently, we transfected Nrf2 overexpression (Nrf2‐OE) plasmids into A549 cells in vitro. Nrf2‐OE resulted in increased p‐Nrf2 and PHB2 expression compared with the negative control (NC) group, which was further enhanced by LTA stimulation (Figure 6F). Mitochondrial and cytoplasmic fractionation of Nrf2‐OE A549 cells showed elevated levels of both mito‐ and cyto‐PHB2 compared with the NC group (Figure 6G). Moreover, the initially decreased expressions of mito‐ and cyto‐PHB2 in the NC group were restored under LTA attack in the Nrf2‐OE group (Figure 6G).

In short, the absence of Nrf2 in SA‐ALI leads to a decrease in mito‐PHB2 levels and an increase in cyto‐PHB2. Conversely, overexpression of Nrf2 promotes the protection of PHB2 against LTA‐induced damage. These findings indicate a positive regulatory role of Nrf2 in modulating PHB2.

### 2.7 Nrf2 binds to the PHB2 promoter and enhances gene transcription

**FIGURE 7:**
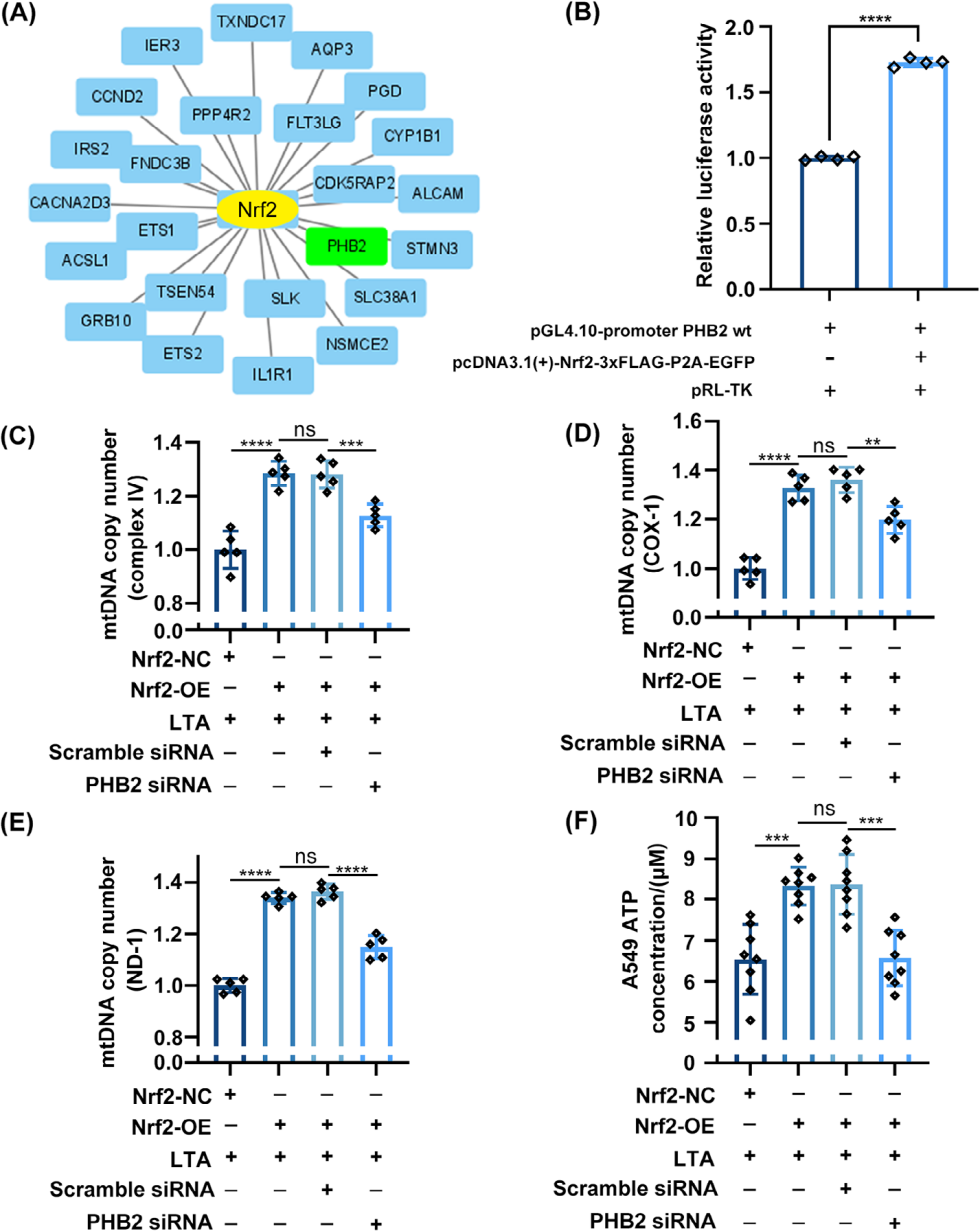
Nrf2 binds to the PHB2 promoter and enhances gene transcription. (A) Input DEGs of GSE95233 into ChEA3 (https://maayanlab.cloud/chea3/) to obtain the downstream of Nrf2. (B) Dual‐luciferase reporter system was used to test Nrf2 binds to the PHB2 promoter region. The thymidine kinase promoter‐renilla luciferase (pRL‐TK) vector was transfected as an internal reference to correct the transfection efficiency. (C–E) The relative levels of complex IV, ND‐1, and COX‐1 were used to reflect the mtDNA copy number. (F) ATP content was measured in A549 cells treated with Nrf2‐OE plasmid and PHB2 siRNA. Experiments were repeated at least three times *p < 0.05, **p < 0.01, ***p < 0.001, ****p < 0.0001, ns denotes not significant.

To determine whether Nrf2 directly enhances PHB2 transcription, we performed a bioinformatic analysis and the dual‐luciferase reporter assay. The 1023 DEGs identified between sepsis and nonsepsis patients (Figure S1C) were input into ChIP‐X Enrichment Analysis Version 3 (ChEA3), a web‐based transcription factor enrichment analysis tool that includes 6 primary reference gene set libraries from multiple sources.^34^ Our research demonstrated the significance of Nrf2 as a crucial transcription factor in sepsis, and notably, Nrf2 exhibited enrichment when PHB2 was employed as input (Figure 7A). In the dual‐luciferase reporter assay, the fluorescence intensity was higher in A549 cells cotransfected with PHB2 wt and Nrf2‐OE luciferase plasmids compared with cells transfected with the former plasmid only (Figure 7B). These results suggest that Nrf2 directly induces PHB2 transcription by binding to its promoter.

To verify whether the direct regulation of PHB2 by Nrf2 indeed affects mitochondrial function, we introduced PHB2 siRNA to inhibit the expression of PHB2. We found that under LTA stimulation, Nrf2‐OE in A549 cells significantly increased the mtDNA copy number. However, after using PHB2 siRNA, the replication process of mitochondrial genes was significantly decreased (Figures 7C–E). However, after using PHB2 siRNA, we observed that although the mtDNA copy number (NADH dehydrogenase subunit 1, ND‐1; cytochrome *c* oxidase subunit I, COX‐1; and complex IV) remained higher than the LTA stimulation group, it was significantly lower than the PHB2 siRNA negative control group (Figures 7C–E). This suggests that in addition to promoting mitochondrial biogenesis and improving mitochondrial function through the expression of downstream mitochondrial protective genes,^17^ Nrf2 can also enhance mitochondrial function by promoting the expression of PHB2. Additionally, we also measured the ATP concentration in A549 cells. Under the influence of LTA, the cellular ATP concentration significantly increased (Figure 7F). Nrf2‐OE further elevated the ATP concentration, but under the interference of PHB2 siRNA, the ATP concentration decreased (Figure 7F). The observed results may be attributed to the following factors: under inflammatory attack, increased intracellular oxidative stress leads to an accumulation of free radicals, stimulating mitochondrial hyperfunction and enhancing OXPHOS efficiency. However, a decrease in ATP concentration was observed when PHB2 siRNA was used in Nrf2‐OE cells (Figure 7F), indicating the loss of the protective role of PHB2 in mitochondrial function and cell survival. This finding indirectly suggests that the Nrf2/PHB2 pathway is involved in maintaining mitochondrial functional homeostasis.

In summary, Nrf2 binds to the PHB2 promoter and directly induces its transcription, as shown by bioinformatics analysis and dual‐luciferase reporter assays. The direct regulation of Nrf2/PHB2 pathway affects mitochondrial function, as evidenced by the introduction of PHB2 siRNA to inhibit PHB2 expression in Nrf2‐OE cell.

## 3 DISCUSSION

This study investigated the involvement of Nrf2 in ALI caused by SA‐sepsis. The main findings can be summarized as follows: (1) Nrf2 was downregulated in the serum of SA‐sepsis patients, which was negatively correlated with severity of sepsis; (2) SA‐ALI was associated with oxidative stress injury and mitochondrial dysfunction, which were exacerbated by loss of Nrf2; (3) Nrf2 modulates PHB2 expression and intracellular distribution during SA‐ALI; and (4) Nrf2 overexpression increased PHB2 expression by directly binding to the PHB2 promoter and activating its transcription. In summary, Nrf2 directly regulates PHB2 expression to maintain mitochondrial function and alleviate SA‐ALI.

Serum Nrf2 levels were found to be decreased in patients with SA‐sepsis, where 81.8% of patients exhibited varying degrees of ALI. Nrf2 protects lung tissue in sepsis by inhibiting inflammation and oxidative stress, maintaining mitochondrial biogenesis, and promoting mitochondrial fusion.^9^, ^35^, ^36^, ^37^ A correlation analysis of serum Nrf2 levels and clinical data from ICU patients revealed that Nrf2 expression was negatively correlated with the degree of organ injury and severity of sepsis. Among the markers commonly used to diagnose sepsis, including PCT and C‐reactive protein (CRP),^38^ we found that the elevated levels of PCT but not CRP in sepsis patients compared with nonsepsis controls, suggesting its greater sensitivity and specificity in detecting bacterial infection during sepsis than CRP.^39^ The PaO_2_/FiO_2_ ratio, which is used to access the severity of ALI or acute respiratory distress syndrome, was found to be lower in SA‐sepsis patients with a lower Nrf2 level under conditions that excluded ventilator‐associated lung injury. By integrating these clinical findings with the results of GO analysis and KEGG pathway enrichment analysis, we can infer that there is a significant occurrence of OXPHOS, cellular energy metabolism, and inflammatory response in sepsis patients. In the animal model, a significant decrease in serum SOD activity was observed in SA‐ALI mice with Nrf2 deficiency. This suggests a significant reduction in the mice's antioxidant capacity. It may lead to the accumulation of free radicals and increased oxidative damage, further exacerbating the inflammatory response and lung injury. Moreover, Nrf2 and its downstream genes have a substantial direct or indirect impact on mitochondrial function, which directs the research focus towards investigating the effects of Nrf2 and mitochondrial functional changes on SA‐ALI. Our results showed a downward trend of mtDNA copy number in Nrf2^−/−^ mice even in the absence of SA‐sepsis, implying that Nrf2 plays an important role in maintaining mitochondrial genome integrity. At the cellular level, total‐Nrf2 expression gradually decreased after treatment with LTA time gradient and concentration, while the opposite was true for p‐Nrf2. That is, in the presence of increased LTA stimulation, the dissociation of Nrf2 from Keap1 and its nuclear translocation is increasing. An increase in the p‐Nrf2/t‐Nrf2 ratio suggests that Nrf2 is becoming more active and is being translocated from the cytoplasm to the nucleus where it can bind to AREs and regulate the expression of target genes involved in the antioxidant response. Data from clinical samples, cell model and an animal model of SA‐sepsis showed that Nrf2 protects against SA‐ALI by maintaining mitochondria function, and is a potential marker for clinical monitoring of sepsis‐induced ALI.

The most notable finding of this study was that Nrf2 deficiency reduced PHB2 expression in SA‐ALI as well as PHB2 translocation from mitochondria to the cytoplasm, leading to mitochondrial dysfunction. PHB2 forms a ring complex with PHB1 on the inner mitochondrial membrane (IMM) and may play a role in activating mitophagy, maintaining mitochondrial genome stability, and regulating mitochondrial fusion; meanwhile, cytoplasmic PHB2 is thought to induce mitochondrial apoptosis.^21^, ^22^ Total PHB2 expression was decreased in the lung tissue of SA‐ALI mice and A549 cells, which was accompanied by impaired mitochondrial function and was exacerbated by the loss of Nrf2. This indicates a possible association between Nrf2 and PHB2, which is supported by the positive correlation between Nrf2 and PHB2 expression observed in a rat subarachnoid hemorrhage model.^25^, ^40^ Our results showed that mito‐PHB2 was decreased while cyto‐PHB2 was increased in lung cells of Nrf2^−/−^ mice, an effect that was enhanced in SA‐ALI, implying that Nrf2 plays a key role in the mitochondrial retention of PHB2. Meanwhile, p‐Nrf2 was maintained at a higher level after Nrf2 overexpressed compared with the negative control group. Combined observation of p‐Nrf2, total‐Nrf2, and total‐PHB2 revealed that LTA attack after Nrf2 overexpressed increased the expression of p‐Nrf2 and total‐PHB2. Based on our observations when Nrf2 is overexpressed, stimulation with LTA significantly increased the mtDNA copy number and ATP concentration. These finding indicates that during sepsis, Nrf2 overexpression leads to an increase in PHB2 expression, thereby protecting mitochondrial function. It agrees with the results of the dual‐luciferase reporter assay in our subsequent experiments. Cyto‐PHB2 has been shown to mediate communication between the nucleus and mitochondria.^41^ It is noteworthy that Nrf2‐OE led to elevated levels of both mito‐PHB2 and cyto‐PHB2 in the presence of LTA. These findings suggest that the upregulation of PHB2 transcription induced by Nrf2 may account for the increased cellular levels of PHB2, as supported by the results of the dual‐luciferase reporter assay. However, the detailed mechanisms underlying the effects of different localization of PHB2 in mitochondria, cytoplasm, and nucleus on mitochondrial function remain to be elucidated. B cell lymphoma 2‐associated X protein inhibitor 1 was shown to mediate PHB2 localization to the outer mitochondrial membrane,^21^ while PHB1 and PHB2 subunits are inserted into the IMM by translocase of the inner membrane 23 where they form a 120‐kDa assembly intermediate^42^ that assembles into large ring complexes on the IMM.^43^ Nuclear localization of PHB2 involves estrogen receptor‐α.^44^ Our data suggest that PHB2 may mediate communication between different cellular organelles. Only a limited number of studies have reported on the Nrf2/PHB2 pathway. Zhang et al.'s^25^ research has found that the Nrf2/PHB2 pathway plays a crucial role in promoting blood–brain barrier protection and reducing neuroinjury in subarachnoid hemorrhage. MitoQ, as a mitochondria‐targeted antioxidant, can enhance the activation of Nrf2 and the expression of PHB2, thereby enhancing cellular antioxidant capacity and improving early brain injury and delayed neurological dysfunction.^40^ This is consistent with our research findings, indicating that Nrf2 positively regulates PHB2, thereby increasing the organism's antioxidant capacity. Although Lai et al.^45^ did not directly investigate the upstream‐downstream relationship between Nrf2 and PHB2, in a Parkinson's disease cell model, the protein expression of PHB2 showed a positive correlation with Nrf2 and was associated with oxidative stress. Various indications suggest that the connection between Nrf2 and PHB2 is universally present and both are implicated in oxidative stress. Our study confirms for the first time the regulatory role of Nrf2 on PHB2 in an inflammation model, and Nrf2 directly promotes the expression of PHB2 by acting on the promoter region.

In the last decade, there have been multiple clinical trials of drugs that activate Nrf2 for the treatment of various diseases. We previously reported that 4‐octyl itaconate, a product of energy metabolism and the immune response with anti‐inflammatory and antioxidant activities in human, prevent ALI in sepsis via the Nrf2/ARE pathway. Sophoricoside and salvinorin A are plant extracts that were shown to alleviate ALI in a Nrf2‐dependent manner.^46^, ^47^ Only four compounds—namely, sulforaphane, dimethyl fumarate, bardoxolone methyl, and oltipraz^48^—have been tested in clinical trials, and none have examined their effects in patients with organ damage secondary to sepsis. The results of the present study provide a reference for the design of clinical studies of Nrf2 activators for the prevention and treatment of SA‐ALI.

Our study has certain limitations that need to be acknowledged. First, due to significant limitations in data collection and volunteer recruitment, it was difficult to include a larger number of suitable patients as controls. This unavoidable limitation may have affected the representativeness of our study. Second, in the SA‐sepsis group, there were patients with mixed bacterial infections, while the control group included patients with different bacterial infections that did not result in sepsis. This introduced systematic errors into our study. Third, due to the voluntary nature of participation in clinical research, some patients who meet the inclusion criteria choose not to participate in this study, may leading to an inevitable selection bias that could affect the representativeness and generalizability of the results. Furthermore, although there were no significant differences in the chronic disease status of patients between the two groups, differences within groups could not be eliminated due to different diseases of patients admitted into ICU. In future studies, we will recruit patients admitted to the ICU with a fixed diagnosis and increase the sample size to increase the persuasiveness of the data. Regarding the choice of animal model, we opted for intraperitoneal injection based on the advantages it offers, such as precise bacterial inoculum, reproducibility of data, and simulation of systemic infection. However, in clinical practice, the initial site of infection in patients with sepsis may not necessarily be an abdominal infection; it could be a pulmonary infection, skin infection, or even a mixed infection. This introduces an inevitable systemic error when extrapolating our animal experimental results. In the process of including clinical cases, we also observed an important phenomenon, namely, not all *S. aureus* sepsis patients had detectable bacteria in their blood cultures. The diagnosis of sepsis is typically based on a comprehensive assessment of clinical features, biomarkers, hemodynamics, tissue perfusion, and organ dysfunction evaluation. According to the recommendations of the Third International Consensus Definitions for Sepsis and Septic Shock (*sepsis‐3*), the diagnosis of sepsis is determined through the assessment of SOFA score and systemic inflammatory response syndrome criteria.^49^ It is important to note that there is currently no gold standard for diagnosing sepsis. Studies have shown that approximately 18% of patients with *S. aureus* sepsis have estimated bacterial concentrations in blood cultures below 0.04 CFU/mL.^50^ Additionally, research has revealed that the average pathogen concentration in the bloodstream of patients with sepsis is only around 0.25 CFU/mL.^51^ Moreover, due to the high‐dose antibiotic therapy commonly administered to these patients, bacterial concentrations in the blood are often low, leading to a significant occurrence of false‐negative results in blood cultures.^52^, ^53^ Therefore, in this study, although positive cultures of *S. aureus* were found in sputum cultures of all sepsis patients, only four cases showed positive results in blood cultures. Our study also has limitations in the mechanistic investigation. We examined the regulatory interaction between Nrf2 and PHB2 but did not explore how mito‐PHB2 is released to the cytoplasm in the context of SA‐ALI. Despite these limitations, our study demonstrates that Nrf2 increased PHB2 expression to prevent mitochondrial damage in ALI associated with SA‐sepsis. These findings suggest that agonists of Nrf2 and PHB2 can be used to mitigate SA‐ALI.

In conclusion, this study elucidated the critical role of Nrf2 in protecting against SA‐sepsis‐associated ALI. The downregulation of Nrf2 in SA‐sepsis patients correlated with the severity of sepsis and lung injury. Nrf2 acted as a key regulator in maintaining mitochondrial function and mitigating oxidative stress in ALI. It directly regulated the expression of PHB2, a protein involved in mitochondrial function, by binding to its promoter and activating transcription. The deficiency of Nrf2 exacerbated mitochondrial dysfunction and oxidative stress in SA‐ALI, highlighting the importance of Nrf2 in preserving lung tissue integrity during sepsis. These findings provide insights into the molecular mechanisms underlying SA‐ALI and suggest that targeting the Nrf2–PHB2 pathway may offer potential therapeutic strategies for sepsis‐associated ALI. Further investigations are warranted to explore the clinical implications and therapeutic potential of modulating Nrf2 and its downstream targets in the management of sepsis and associated lung complications.

## 4 MATERIALS AND METHODS

### 4.1 Patients and ethics

Serum samples were collected from patients diagnosed with SA‐sepsis (*n* = 11) and nonsepsis control patients (*n* = 22) at the ICU of Wuxi Hospital of Traditional Chinese Medicine and Affiliated Hospital of Jiangnan University during the period from March 1, 2019 to August 31, 2020. All sepsis patients were enrolled consecutively. Whole blood and clinical data were collected from patients after diagnosis as sepsis within 24 h of their admission to the ICU. Sepsis was defined according to *Sepsis‐3*.^49^ We obtained the APACHE II score^54^, ^55^ and the SOFA score^56^, ^57^ of each patient—which revealed the severity of organ dysfunction during sepsis—by examining clinical data and laboratory indices (Table S1). The exclusion criteria were age <18 years; history of immune deficiency diseases or malignancy; inability or refusal to provide written, informed consent; being in a terminal state of sepsis; or having undergone/undergoing chemotherapy or hormone therapy within 4 weeks of ICU admission. Medical and nursing records were reviewed to collect patient demographics, ventilator parameters, and laboratory findings (Table 1).

All patients or their families expressed willingness to participate in the study through provision of signed, informed consent. Experiments involving human subjects were approved by the Ethics Committee of the Affiliated Hospital of Jiangnan University, Wuxi, China (ethics no. LS2020038).

Peripheral venous blood samples were collected from patients using a vacuum blood collection tube without anticoagulant. After they had been allowed to stand for 30 min, the samples were briefly centrifuged and the supernatant was aspirated, followed by centrifugation (6800 *g*, 10 min). The serum was stored at −80°C. Serum Nrf2 level was determined using a commercial ELISA kit (Abcam; Cat. No: ab277397).

### 4.2 RNA‐sequencing dataset

The GSE95233 dataset from the Gene Expression Omnibus (GEO) database (https://www.ncbi.nlm.nih.gov/geo/; derived from the GPL570 [HG‐U133_Plus_2] Affymetrix Human Genome U133 Plus 2.0 Array) included 22 control patients and 51 patients with septic shock; the latter underwent two blood draws, yielding 102 samples in total. We compared the 102 samples from septic shock patients (from GSM2500371 to GSM2500472) to control samples (from GSM2500349 to 2500370) and used the “limma” package of R software (R Foundation for Statistical Computing, Vienna, Austria) to identify DEGs between sepsis patients and controls.

### 4.3 GO and KEGG pathway enrichment analyses

We used the “ClusterProfiler” package of R software^58^ to perform GO and KEGG pathway enrichment analyses to determine the molecular functions and signaling pathways of the identified DEGs.

### 4.4 Reagents and antibodies

LTA was purchased from Sigma–Aldrich (Cat. No: L3140). ML385 (Cat. No: HY‐100523), a Nrf2 inhibitor, was from MedChemExpress. Cell nuclei were stained with 4′,6‐diamidino‐2‐phenylindole (Beyotime; Cat. No: C1018) or Hoechst33342 (Yeasen; Cat. No: 40732ES03). Antibodies used in this study are listed in Table S2.

### 4.5 Cell culture and treatment

A549 lung epithelial cells were purchased from the American Type Culture Collection (ATCC) and cultured in Dulbecco's Modified Eagle's Medium supplemented with 10% fetal bovine serum and 1% penicillin‐streptomycin solution (all from Gibco; Cat. No: 16140071 and 11965092) at 37°C with 5% CO_2_. A reserve solution of ML385 with a concentration of 10 mM was dissolved in DMSO. 5 mg of LTA was dissolved in 500 μL of ultrapure water. In the LTA time gradient experiment, cells were treated with a culture medium containing 10 μg/mL of LTA and incubated for 0.5, 2, and 4 h, respectively. In the LTA concentration gradient experiment, cells were treated with a culture medium containing 2.5, 5, 10, and 20 μg/mL of LTA, respectively, and incubated for 4 h. In the MMP and western blotting experiments, cells were pre‐treated with ML385 at a concentration of 10 μM for 0.5 h and then exposed to 20 μg/mL of LTA for 4 h.^59^

### 4.6 Bacterial strains and growth conditions

Bacterial cell recovery and preservation were performed as previously described.^60^ Briefly, lyophilized powder of *S. aureus* strain ATCC29523 was cultured overnight (37°C, 220 rpm) to mid‐log phase (optical density = 600 nm) in brain heart infusion‐chloramphenicol broth. The cell suspension was evenly spread on nonselective Luria Broth medium for single colony counting. The bacterial solution was mixed with 40% glycerol in a 1:1 ratio and stored at −80°C.

### 4.7 Animals and ethics

Animal experiments were approved by the Animal Ethics Committee of Jiangsu Institute of Schistosomiasis Control (ethics no. IACUC‐JIPD‐2019043). WT C57BL/6 mice (8 weeks old, weighing 18−25 g) were obtained from Jiangnan University (Wuxi, China). Male Nrf2^−/−^ mice (8 weeks old, weighing 18−25 g) were obtained from the Model Animal Research Center (Nanjing, China).^61^ Genotyping (Figure S4B) and RT‐qPCR (Figure S4C) were performed to confirm Nrf2 deficiency. The SA‐ALI model was established by intraperitoneal injection of *S. aureus* into 8‐week‐old mice.^7^, ^62^ Specific amounts of SA were suspended in a suitable solution and then injected into the abdominal cavity of mice using a syringe, avoiding blood vessels, bladder, and any viscera.

WT and Nrf2^−/−^ mice were divided into control (100 μL of phosphate‐buffered saline [PBS] was injected intraperitoneally), SA1 (2 × 10^7^ CFU *S. aureus* dissolved in 100 μL PBS), SA2 (1 × 10^8^ CFU *S. aureus* dissolved in 100 μL PBS), and SA3 (3 × 10^8^ CFU *S. aureus* dissolved in 100 μL PBS) groups according to the concentration of *S. aureus* that was injected.^63^, ^64^ After intraperitoneal injection of PBS/SA suspension in mice for 6 h,^65^ inhale anesthesia with a dose of 2.0% isoflurane for 30 min. After 6 h of intraperitoneal injection of PBS/SA in mice, the mice were anesthetized and dissected to obtain whole blood, serum, BALF, fresh lung tissue, and paraformaldehyde‐fixed lung tissue. Immediately after anesthesia, collect 0.8–1 mL of whole blood from the posterior vena cava. After a 30‐min stasis, follow the steps in section 4.11 to obtain the serum. Simultaneously, open the airway and chest of the mice and use the method described in section 4.8 to obtain BALF. Within 20 min after anesthesia, collect fresh lung tissue from the mice. Store a portion at −80°C and fix another portion in 4% paraformaldehyde at room temperature. The paraformaldehyde‐fixed lung tissue will be paraffin‐embedded within 48 h. The portion of the fresh lung tissue is used for western blot and qRT‐PCR. Bacterial cultures of BALF and peripheral whole blood confirmed that the sepsis model was successfully established (Figure S4A).

### 4.8 BALF

After tracheotomy, the alveoli of mice were lavaged using sterile pre‐chilled PBS. The BALF was centrifuged and protein concentration in the supernatant was determined using a bicinchoninic acid assay protein quantification kit (Vazyme; Cat. No: E112‐01). The pellet was used for cell counting; the total number of cells in BALF was determined by flow cytometry (BD C6; BD Biosciences).

### 4.9 RT‐qPCR

RNA was extracted from lung tissue or cells using TRIzon reagent (CWBIO; Cat. No: CW0580) according to the manufacturer's instructions. The PrimeScript RT kit (Takara; Cat. No: RR047A) and SYBR Mix (Yeasen; Cat. No: 11198ES08) were used for reverse transcription and qPCR, respectively. Primer sequences and PCR conditions are listed in Tables S3 and S4. Expression levels of each gene were normalized to glyceraldehyde 3‐phosphate dehydrogenase (GAPDH) and expressed as a fold of control. Observe the dissociation curve for the presence of only a single amplification peak to verify reaction specificity. This experiment was performed using LightCycler 480 Real Time PCR System (Roche). The 2^−∆∆Ct^ method was used to quantify the reaction mtDNA transcriptional activity was measured by examining the expression of ND‐1, COX‐1, and complex IV.

### 4.10 Histopathology and lung injury score

Mouse lung specimens were fixed in 4% paraformaldehyde for 48 h and then embedded in paraffin (ASP200S and EG1150H; Leica). Paraffin blocks were cut into 4 μm thick sections on a microtome (RM2245; Leica) that were imaged under a pathology scanner (Pannoramic MIDI; 3DHISTECH) after H&E staining. The lung injury score was assigned according to American Thoracic Society criteria based on examination of five randomly selected fields by two pathologists who were not involved in the study. The average score of the five random regions is taken as the lung injury score of this mouse, which is represented by this slice.

### 4.11 Serum biochemical indicators

After collecting whole blood from mice, the samples were placed in 1.5 mL EP tubes and allowed to stand for 30 min to facilitate the separation of plasma and serum. The samples were centrifuged for 10 min at 425 *g* and then for 20 min at 106 *g*. The supernatant was carefully aspirated to obtain 300 μL of serum at least, which was immediately stored at −80°C. The concentration of serum LDH (Cat. No: A020‐2‐2), MDA (Cat. No: A003‐1‐2), SOD (Cat. No: A001‐3‐2), and MPO (Cat. No: A044‐1‐1) was measured using commercial kits from Nanjing Jiancheng Bioengineering Institute (Nanjing, China).

### 4.12 Western blotting

Total protein from lung tissues and cells was extracted and western blotting was performed as previously described.^60^ Briefly, cells and tissue extracts were resolved by 10 or 12% SDS‐PAGE, transferred to nitrocellulose membrane, and incubated using various primary antibodies (Table S2) diluted 1:1000 at 4°C overnight, then incubated for 1 h at room temperature with the corresponding HRP‐conjugated secondary antibody. Protein bands were visualized and imaged using ECL Chemiluminescence Assay Kit (Vazyme; Cat. No: E412‐01) and Gel Image Analysis System (Universal Hood III; Bio‐Rad). After importing the image into ImageJ software (National Institutes of Health, Bethesda, MD, USA), the gray values of bands were calculated after removing the background signal. VDAC1 was used as the loading control for mitochondrial proteins and GAPDH or β‐actin was used as the loading control for whole‐cell and cytoplasmic proteins.

### 4.13 Mitochondrial isolation

Fresh mouse lung tissue (0.1 g) or cells (5 × 10^6^) were collected and homogenized on ice and lysed to obtain intact and purified mitochondria using the Mitochondria Isolation kit (Bioss; Cat. No:C5032) according to the manufacturer's protocol.

### 4.14 Measurement of cellular ATP concentration and cell viability

Cell viability was assessed using Cell Counting Kit‐8 (Vazyme; Cat. No: A311‐01) and cellular ATP concentration was measured using the Enhanced ATP Assay kit (Beyotime; Cat. No: S0027), both according to the manufacturer's instructions.

### 4.15 MMP assay

The MMP assay was performed using a kit (Solarbio Science Amp Technology; Cat. No: M8650) according to the manufacturer's instructions. Fluorescence was visualized by confocal laser scanning microscopy at 400 × magnification (Model LSM880; Carl Zeiss).

### 4.16 Plasmid construction and transfection

The Nrf2‐OE plasmid pcDNA3.1(+)‐Nrf2‐3 × FLAG‐P2A‐EGFP (forward primer [CMV‐F], CGCAAATGGGCGGTAGGCGTG; reverse primer [EGFP‐SEQR], GACACGCTGAACTTGTGGC) and the control vector H2713 pcDNA3.1(+)−3 × FLAG‐P2A‐EGFP were designed and constructed by OBiO Technology. A549 cells were seeded at a density of 5 × 10^5^ cells per well in six‐well plates and transfected with 2 μg DNA per well mixed with 5 μL Liposomal Transfection Reagent (Yeasen; Cat. No: 40802ES02) according to the manufacturer's protocol.

### 4.17 Immunofluorescence analysis

Immunocytochemistry was performed as previously described.^66^ Fluorescence was visualized by confocal laser scanning microscopy at 400 × magnification and analyzed using ZEN2 (blue edition) software (Carl Zeiss).

### 4.18 Dual‐luciferase reporter assay

The PHB2 luciferase plasmid pGL4.10‐promoter PHB2 wt (forward primer [RVprimer3], CTAGCAAAATAGGCTGTCCC; reverse primer [Luc2‐N‐Re], CGTCTTCGAGTGGGTAGAATG) and control vector H352 pGL4.10 were constructed by OBiO Technology.

The PHB2 luciferase plasmid and Nrf2‐OE plasmid were cotransfected into cells and the dual‐luciferase reporter assay was performed using a kit (Vazyme; Cat. No: DL101‐01) according to the manufacturer's protocol. Fluorescence was detected using a microplate reader (SynergyH4; Berthold Technologies).

### 4.19 PHB2 RNA silencing

To enhance gene silencing efficiency, a mixture of three different PHB2 siRNAs was used: 5′‐GCCACATCACAGAATCGTA‐3′; 5′‐GAACCCTGGCTACATCAAA‐3′; 5′‐CCAAGGACTTCAGCCTCAT‐3′. The siRNAs were commercially synthesized (RiBobio). For negative controls, scrambled RNA oligonucleotides were used. For each well, 50 nM of each of the three oligos were transfected using Hieff Trans™ Liposomal Transfection Reagent (Yeasen, Cat. No: 40802ES02) according to the manufacturer's instructions. After incubation for 15 min, the medium was replaced with DMEM containing 10% fetal bovine serum. Transfected cells were used for the subsequent experiments 48 h after transfection.

### 4.20 Statistical analysis

Data are presented as means ± standard deviation. The difference between the two groups was evaluated for significance with an unpaired t‐test. Continuous variables in clinical data were tested for normality. The independent samples *t*‐test and Wilcoxon rank‐sum test were applied to normal and non‐normal data, respectively. The chi‐squared test was used to evaluate categorical variables in clinical data. Correlations were determined by linear regression analysis. Cumulative survival rates were determined with the log‐rank test. A *p* value < 0.05 was taken to reflect a significant difference in all tests. Prism v9.0 (GraphPad), Cytoscape v 3.8.2 (https://cytoscape.org/release_notes_3_8_2.html), SPSS v20 (IBM), R v.4.1.1, and Bioconductor v3.13 (https://bioconductor.org/news/bioc_3_13_release/) were used for data analysis and image processing.

## AUTHOR CONTRIBUTION

Dr. Zhiqiang Wang, Yaxian Wu, and Qingfeng Pang conceived and designed the experiments. Sihao Jin and Jiaojiao Sun are the major contributors of this manuscript and contributed equally to this article. Drs. Lijuan Shen, Yuan Weng, and Min Chen analyzed and interpreted the patient data regarding the sepsis. Sihao Jin, Jiaojiao Sun, Yingying Wang, and Zhiqi Gao performed the experiments. Sihao Jin and Gang Liu analyzed the bioinformatics data. Fengjuan Jiang, Jinyou Li, and Shengpeng Li checked the clinical data. Drs. Qingfeng Pang, Zhiqiang Wang, Yaxian Wu, and Dan Chen revised the article. All authors read and approved the final manuscript.

## CONFLICT OF INTEREST STATEMENT

The authors have disclosed that they do not have any potential conflict of interest.

## ETHICS STATEMENT

All subjects signed on written informed consent, and human subject approval was obtained from the Ethics Committee of the Affiliated Hospital of Jiangnan University, Wuxi, China (ethics no. LS2020038). All animal experimental procedures were approved by the Animal Ethics Committee of Jiangsu Institute of Schistosomiasis Control (ethics no. IACUC‐JIPD‐2019043).

## Supporting information

Supporting information

## Data Availability

All data generated or analyzed during this study are included in this published article and its supplementary information files. Raw data can be obtained from the corresponding authors.
