# Supplementary material for: Nrf2/PHB2 alleviates mitochondrial damage and protects against Staphylococcus aureus‐induced acute lung injury
Source: MedComm (2020). 2023 Dec 7;4(6):e448. doi: 10.1002/mco2.448 (PMC10701464; doi:10.1002/mco2.448)
Supplement: Supplementary file 1 — Supporting information [file MCO2-4-e448-s001.docx]

# Nrf2/PHB2 alleviates mitochondrial damage and protects against *Staphylococcus aureus*-induced acute lung injury

# Si-hao Jin[^1^](https://pubmed.ncbi.nlm.nih.gov/33987410/#affiliation-1)^,^[^2^](https://pubmed.ncbi.nlm.nih.gov/33987410/#affiliation-2)^,3,#^ , Jiao-jiao sun[^1^](https://pubmed.ncbi.nlm.nih.gov/33987410/#affiliation-1)^,^[^2^](https://pubmed.ncbi.nlm.nih.gov/33987410/#affiliation-2)^,#^, Gang Liu^4^, Li-juan Shen^5^, Yuan Weng^1^, Jin-you Li^1^, Min Chen^6^, Ying-ying Wang[^2^](https://pubmed.ncbi.nlm.nih.gov/33987410/#affiliation-2), Zhi-qi Gao[^2^](https://pubmed.ncbi.nlm.nih.gov/33987410/#affiliation-2), Feng-juan Jiang^2^, Sheng-peng Li[^2^](https://pubmed.ncbi.nlm.nih.gov/33987410/#affiliation-2), Dan Chen[^2^](https://pubmed.ncbi.nlm.nih.gov/33987410/#affiliation-2), Qing-feng Pang[^2^](https://pubmed.ncbi.nlm.nih.gov/33987410/#affiliation-2), Ya-xian Wu^2,*^, Zhi-qiang Wang^1,*^

1 Department of Cardiothoracic Surgery, Affiliated Hospital of Jiangnan University, Wuxi, China.

2 Wuxi School of Medicine, Jiangnan University, Wuxi, China.

3 School of Medicine, Shaoxing Vocational & Technical College, Shaoxing, China.

4 Department of Nosocomial Infection, The Forth Affiliated Hospital of Zhejiang University, Jinhua, China

5 Department of Critical Care Medicine, Wuxi Hospital of Traditional Chinese Medicine, Wuxi, China.

6 Department of Laboratory, Affiliated Hospital of Jiangnan University, Wuxi, China.

# Supplementary Tables

## Table S1. Sepsis-3 criteria for sepsis and septic shock

|  | **Clinical indicators** |
| --- | --- |
| **Sepsis**  **SOFA**  **score** | **Respiration PaO_2_/FiO_2_ (mmHg)**  < 400  < 300  < 200  < 100 |
|  | **Central nervous system (GCS)**  14-13  12-10  9-6  5-3 |
|  | **Renal Creatinine mg/dL (mmol/L) or urine output**  1.2-1.9 (110-170)  2.0-3.4 (171-299)  3.5-4.9 (300-440) or <500 ml/day  ≥5.0 (>440) or <200 ml/day |
|  | **Liver Bilirubin mg/dL (mmol/L)**  1.2-1.9 (20-32)  2.0-5.9 (33-101)  6.0-11.9 (102-204)  >12 (>204) |
|  | **Coagulation platelets ×10^3^/mm^3^**  <150  <100  <50  <20 |
|  | **Cardiovascular Hypotension**  Mean arterial pressure <70 mmHg  Dobutamine (any dose)  Norepinephrine < 0.1 µg/kg/min  Norepinephrine > 0.1 µg/kg/min |
| **Septic**  **shock** | Sepsis and vasopressor therapy needed to maintain MAP ≥65 mmHg and lactate > 2 mmol/L (18 mg/dl) despite adequate fluid resuscitation |

GCS, Glasgow Coma Scale; SOFA, Sequential Organ Failure Assessment; Adapted from Singer M, Deutschman CS, Seymour CW, et al: The third international consensus definitions for sepsis and septic shock (sepsis-3). *JAMA* 2016; 315(8):801-810.

## Table S2. Antibody information

| **Name** | **Manufacturer** | **Catalogue number** |
| --- | --- | --- |
| Cytochrome c | #11940 | Cell Signaling technology (USA) |
| Nrf2 | #12721 | Cell Signaling technology (USA) |
| PHB2 | 66424-1-Ig | Proteintech (China) |
| VDAC1 | 10886-1-AP | Proteintech (China) |
| β-actin | sc-130065 | Santa Cruz Biotechnology (USA) |
| GAPDH | ab8245 | Abcam (USA) |
| p-Nrf2 (S40) | Abs137005 | Absin (China) |

## Table S3. Primer for RT-qPCR in mice

| **Gene** | **Forward prime (5’ to 3’)** | **Reverse prime (5’ to 3’)** |
| --- | --- | --- |
| GAPDH | CCTCGTCCCGTAGACAAAATG | TCTCCACTTTGCCACTGCAA |
| COX-1 | GAAGAGACAGTGTTTCATGTGGTGT | TCCTGGGCCTTTCAGGAATA |
| ND-1 | ATGGTCAGTCTGTCATGGTGGAAC | GCATAGCACAAGCAGCGACAAC |
| Complex IV | CAGGATTCTTCTGAGCGTTCTATCA | AATTCCTGTTGGAGGTCAGCA |
| IL-6 | ACTTCCATCCAGTTGCCTTCTTGG | TTAAGCCTCCGATTGTGAAGTG |
| TNF-α | AGGTTCTCTTCAAGGGACAA | GACTTTCTCCTGGTATGAGATAG |
| IL-1α | TCTATGATGCAAGCTATGGCTCA | CGGCTCTCCTTGAAGGTGA |
| IL-10 | CTTACTGACTGGCATGAGGATCA | GCAGCTCTAGGAGCATGTGG |
| NRF1 | TATGGCGGAAGTAATGAAAGACG | CAACGTAAGCTCTGCCTTGTT |
| PGC-1α | TATGGAGTGACATAGAGTGTGCT | CCACTTCAATCCACCCAGAAAG |
| TFAM | ATTCCGAAGTGTTTTTCCAGCA | TCTGAAAGTTTTGCATCTGGGT |
| OPA1 | AAGTGACAAGCATTACAGG | CTCCAAGATCCTCTGATACT |
| MFN1 | TAATGGCAGAAACGGTAT | TTCCTGTATGTTGCTTCA |
| Nrf2 | AAAATCATTAACCTCCCTGTTGAT | CGGCGACTTTATTCTTACCTCTC |
| PHB2 | CCATTGTTAATGAGGTGCTCAA | CTTCGGATCAACAGGGACA |

## Table S4. RT-qPCR condition

| **Stage** | **Temperature/**℃ | **Time/s** | **Cycles/n** |
| --- | --- | --- | --- |
| Denaturation | 95 | 300 (heating rate 4.4℃/s) | 1 |
| PCR | 95 | 10 (heating rate 4.4℃/s) | 45 (single) |
|  | according to the Tm of primer | 30 (heating rate 2.2℃/s) |  |
| Extension | 95 | 5 | 1 |
|  | 60 | 60 |  |
|  | 95 | continuous |  |
| Cooling | 50 | 30 | 1 |

# Supplementary Figures

## Figure S1


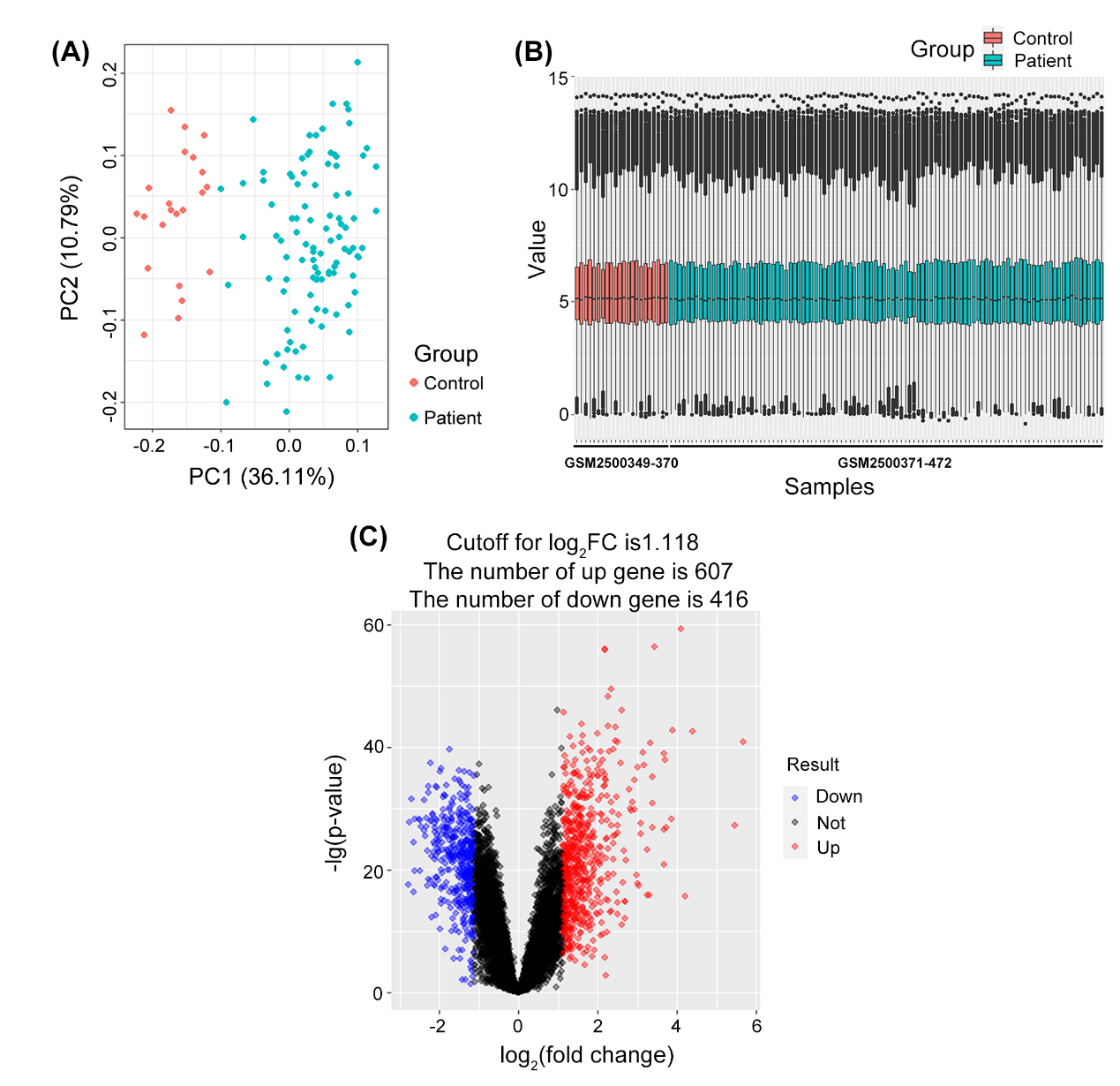


**Figure S1. Principal component analysis (PCA) and gene normalization.** (A) Dimensionality reduction of pooled replicate RNA-seq data using PCA. Control and septic patients can be well distinguished. (B) Normalized the gene expression of each sample revealing that the mean expression was at the same level in both groups. (C) Differential genes in the dataset GSE95233 were filtered out to obtain a volcano plot (|log2 FoldChange|=1.118, p < 0.05).

## Figure S2


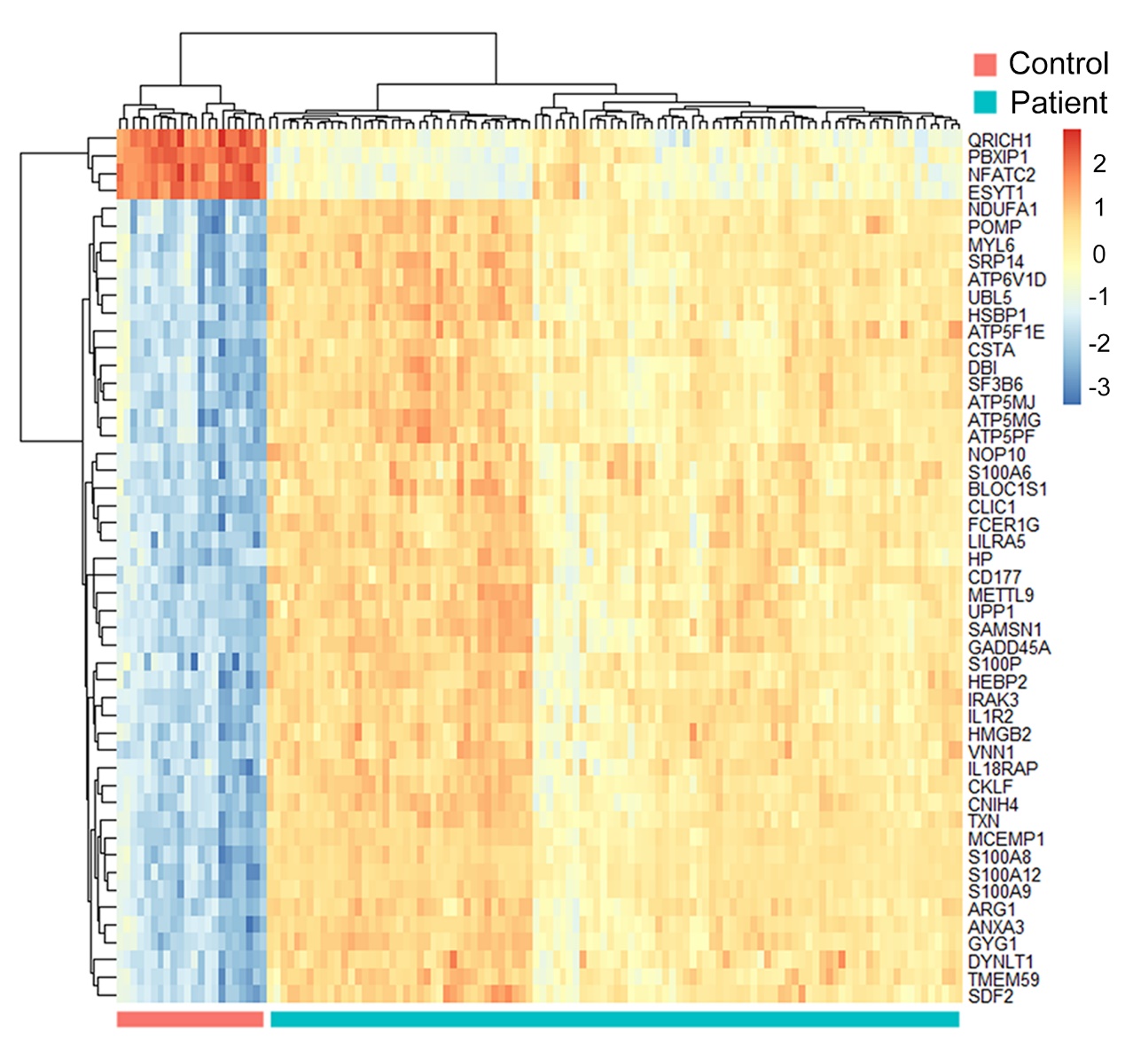


**Figure S2: Differentially expressed genes (DEGs).** DEGs in the dataset GSE95233 were filtered out to obtain a heat map (top 50).

## Figure S3


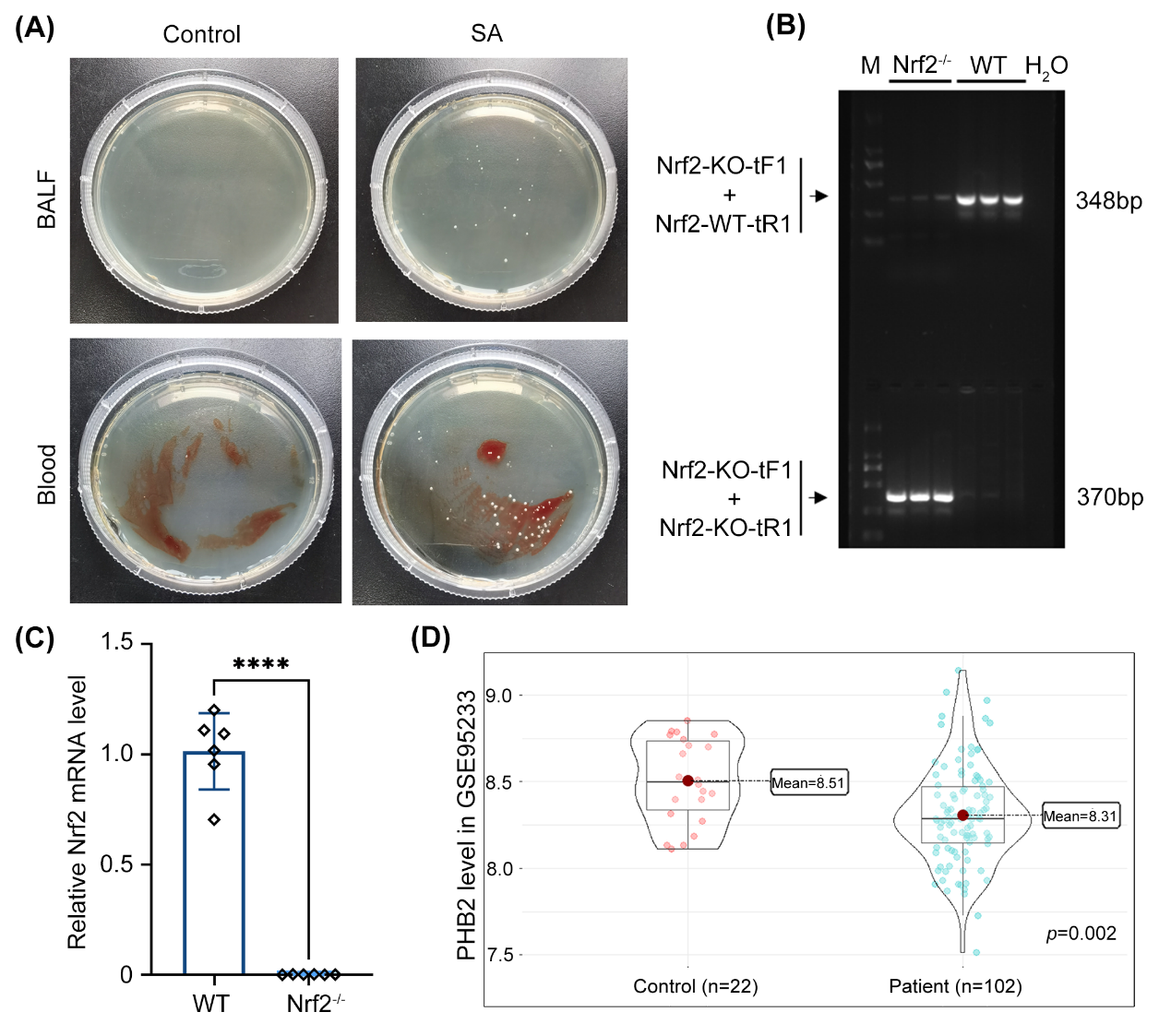


**Figure S3. Model establishment *in vivo* and PHB2 level in patients.** (A) BALF and whole blood from WT mice were evenly spread on solid medium and incubated at 37 ° C for 12 hours. (B) Tails from WT and Nrf2^-/-^ mice were genotyped. (C) Nrf2 level in Nrf2^-/-^ mice was detected by RT-qPCR. C. The gene expression of PHB2 in 22 healthy samples and 102 sepsis samples were obtained from GSE95233. ****p<0.0001.

## Figure S4


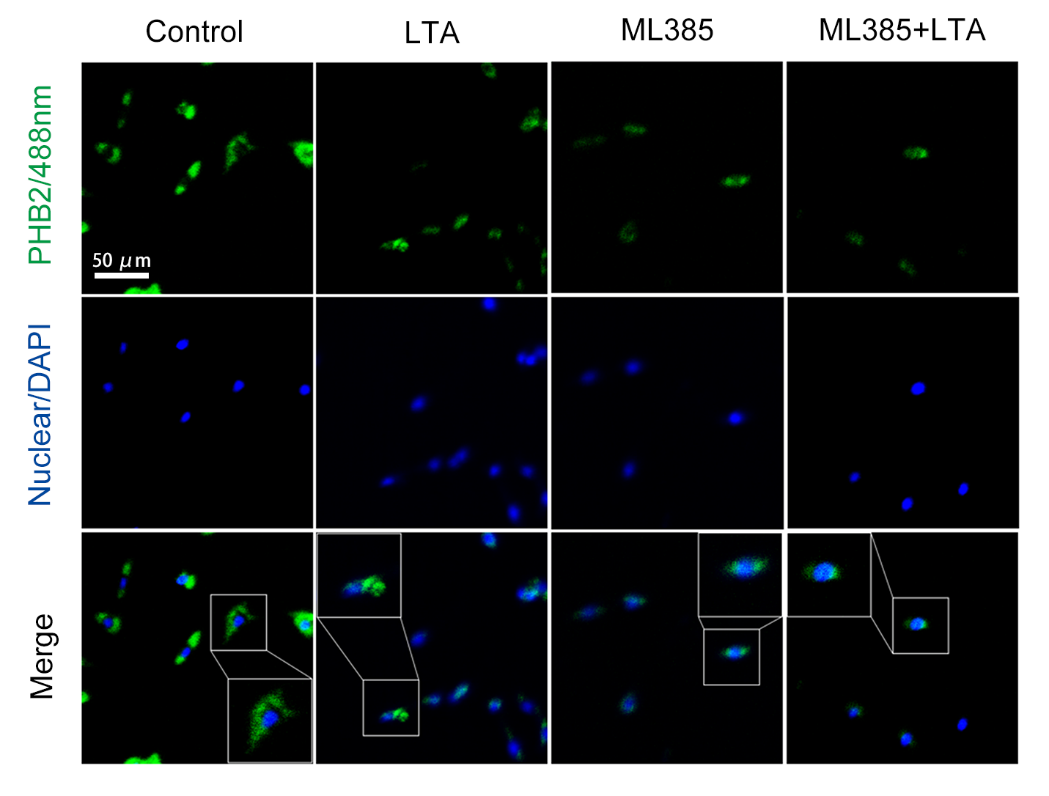


**Figure S4. Nrf2 inhibition assay.** Immunofluorescence analysis in A549 cells were imaged using inverted laser confocal microscope (magnification, ×400, bar =50 µm; n=3).
